# Oat Beta-Glucans Modulate the Gut Microbiome, Barrier Function, and Immune Responses in an In Vivo Model of Early-Stage Colorectal Cancer

**DOI:** 10.3390/ijms252413586

**Published:** 2024-12-19

**Authors:** Magdalena Guzowska, Katarzyna Dziendzikowska, Łukasz Kopiasz, Małgorzata Gajewska, Jacek Wilczak, Joanna Harasym, Malwina Czerwińska, Joanna Gromadzka-Ostrowska

**Affiliations:** 1 Department of Physiological Sciences, Institute of Veterinary Medicine, Warsaw University of Life Sciences, 02-776 Warsaw, Poland; malgorzata_gajewska@sggw.edu.pl (M.G.); jacek_wilczak@sggw.edu.pl (J.W.); 2 Department of Dietetics, Institute of Human Nutrition Sciences, Warsaw University of Life Sciences, 02-776 Warsaw, Poland; lukasz_kopiasz@sggw.edu.pl (Ł.K.); malwina_czerwinska@sggw.edu.pl (M.C.); joanna_gromadzka-ostrowska@sggw.edu.pl (J.G.-O.); 3 Department of Biotechnology and Food Analysis, Wroclaw University of Economics and Business, 53-345 Wroclaw, Poland; joanna.harasym@ue.wroc.pl

**Keywords:** claudins, colon microbiome, colorectal cancer, intestinal barrier integrity, lymphocytes, oat beta-glucan, rats

## Abstract

Oat beta-glucans (OBGs) are known for their beneficial effects on gut health, including anti-inflammatory and prebiotic effects. The aim of this study was to evaluate the impact of two doses (1% or 3% *w*/*w*) of dietary low-molar-mass OBG supplementation on colorectal cancer (CRC) development, immune cell profiles, intestinal barrier protein expression, and microbiota composition in a rat model of CRC induced by azoxymethane (AOM). Microbiome analysis revealed significant differences between the control and CRC groups. OBG supplementation influenced microbial diversity and abundance, particularly increasing the population of beneficial bacteria, such as *Lachnospiraceae* and *Ruminococcaceae*, associated with butyrate production. However, higher doses of OBG (3%) led to a decrease in butyrate-producing bacteria and a shift toward higher levels of *Akkermansia muciniphila* and *Enterococcus faecalis*. Immune cell profiling showed a higher percentage of T lymphocytes (CD3+) in rats fed a diet supplemented with 3% OBG, both in the intraepithelial (IEL) and lamina propria lymphocytes (LPLs). Immunohistochemical analysis of the large intestine revealed a significantly elevated expression of intestinal barrier proteins, i.e., claudin 3 and 4 in rats receiving 1% OBG, while claudin 7 expression was reduced in early-stage CRC. Gene expression analysis also revealed a significant downregulation of *Cldn1* in CRC rats. These findings suggest that dietary OBG supplementation modulates the gut microbiota, immune response, and intestinal barrier integrity, with potential implications for nutritional CRC development prevention and treatment strategies.

## 1. Introduction

Colon cancer, also known as colorectal carcinoma (CRC), is one of the most common gastrointestinal tract malignancies. Epidemiological data show a growing number of new cases and cancer-related deaths, particularly in Europe, the United States, and other developed countries [1,2]. CRC is the third most common cancer worldwide, with over 1.9 million new cases and 930,000 deaths estimated in 2020 [3,4]. The global incidence has more than doubled since 1990, reaching 2.17 million cases in 2019 [4,5]. Incidence rates are highest in Australia/New Zealand and European regions (40.6 per 100,000 males) [6]. Alarmingly, the burden of CRC is projected to increase to 3.2 million new cases and 1.6 million deaths by 2040, with most cases expected to occur in high or very high-Human Development Index countries [7]. While incidence rates are decreasing among individuals aged 50 years and older due to improved screening and early detection, there is a concerning rise in CRC incidence among those younger than 50 years, with an annual increase of approximately 2%, highlighting a significant public health challenge [8].

CRC, a malignancy originating from the epithelial cells of the large intestine, is closely associated with the immune environment of the gut. The interactions between immune cells, intestinal barrier proteins, and the gut microbiota play a pivotal role in both the onset and progression of the disease. The immune cells within the lamina propria and epithelium, along with intestinal barrier proteins, form a complex immune environment that works closely with the intestinal microbiota, forming one of the largest immune organs in the body. The health of this system is influenced particularly by conditions such as inflammatory bowel disease and cancer [9]. Disruptions in this system are strongly linked to CRC, where immune cells in the gut wall, tight junction proteins, and the gut microbiome all contribute to the pathogenesis and progression of the disease [10].

Under physiological conditions, the immune system supports the growth of beneficial bacteria, helping maintain a stable and symbiotic gut environment. In return, these bacteria secrete byproducts (products of their metabolism) that are used both as nutrients for intestinal cells and play an important role in the development and regulation of the intestinal immune system. However, dysbiosis—disruption in the microbiome composition—can lead to various diseases, including cancer. In this altered bacterial flora, cancer-promoting bacteria such as *Enterococcus faecalis* (*Enterococcus faecalis*) and *Allobacullum* sp. often develop, while populations of protective bacteria diminish [11].

Various dietary factors further influence the interaction between the intestinal microbiome and the immune system, with dietary fibre playing a vital role. Fermentative bacteria in the gut convert dietary fibres into short-chain fatty acids (SCFAs), which not only support the integrity of the epithelial barrier but also stimulate immune system function and help prevent inflammation [12].

Beta-glucans, a group of polysaccharides found in microorganisms, sea algae, fungi, and cereals such as barley and oat, have shown potential health benefits. In the previous animal study, we showed that the dietary administration of oat beta-glucan exerts beneficial anti-inflammatory [13], antioxidative [14,15], and prebiotic effects [13]. Additionally, our results indicate that these effects depend on the molar mass of beta-glucan [13,15,16], with more pronounced effects observed in the low-molar-mass fraction (OBG). Therefore, in the presented study, we used low-molar-mass OBG at two doses, 1% and 3% (*w*/*w*), to investigate its potential anti-carcinogenic impact during the early stages of CRC in a rat model.

## 2. Results

### 2.1. Body Weight Gain, Health Status, Food Intake, and Histological Examination of the Large Intestine

There were no significant differences in feed consumption or body weight gain of the rats. Clinical observations of the animals, including appetite, stool consistency, and signs of anal inflammation, did not show any symptoms of pathology, as described in detail in our previous study [17].

A histopathological examination of the large intestine in CRC rats without dietary intervention showed marked changes in the mucosal layer, typical for the early stage of carcinogenesis. These included the presence of aberrant crypt foci, hyperplasia, and erosion, with no visible macroscopic changes indicative of the development of more advanced stages of neoplasia, such as the polyps. In contrast, the control groups and rats with CRC development receiving the OBG displayed normal large intestine histology without early microscopic signs of colorectal cancer. Additionally, there were also no significant differences in the length and width of the inferior, superior, and middle parts of the crypt’s parts between the group with early-stage CRC without dietary intervention and the other groups, as previously described in our article [17].

### 2.2. The Composition of Large Intestine Microbiota—Bacterial Abundance, Diversity, and Taxonomic Analysis

**Figure 1:**
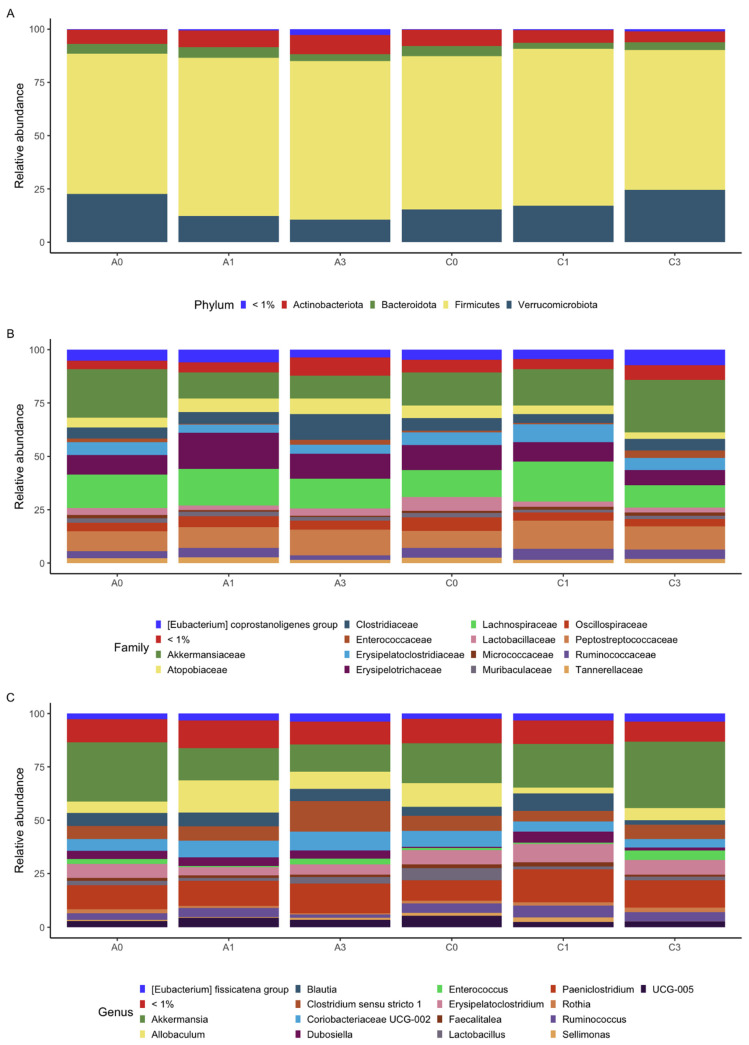
Microbiota composition at the taxonomic class levels of genus (**A**), phylum (**B**), and family (**C**). Cumulated bar plots represent cumulative microbiota composition in each group; C0—control group of rats fed a diet without OBG (n = 7); C1—control group of rats fed a diet supplemented with 1% OBG (n = 6); C3—control group of rats fed a diet supplemented with 3% OBG (n = 5); A0—CRC model rats group fed a diet without OBG (n = 8); A1—CRC model rats group fed a diet supplemented with 1% OBG (n = 7); A3—CRC model rats group fed a diet supplemented with 3% OBG (n = 7).

We attempted to assess the intestinal microbiome profiles in a control group (group C) and animals with AOM-induced CRC (group A), as well as its dependence on dietary supplementation with OBG (at a dose of 1% and 3%) by sequencing a fragment of the 16S rRNA gene (V3–V4 variable region). The total number of reads in the sequenced samples ranged from 102,489 to 200,978. DADA2 analysis resulted in 532 ASVs after denoising and singleton removal. These obtained ASVs were compared with sequences contained in the SILVA database, using its phylogenetic classification standards (https://www.arb-silva.de/browser/ssu/, accessed 30 October 2024 [18]. In total, seven phyla, 11 classes, 28 orders, 42 families, and 108 genera were identified. Of the detected ASVs, 10 could not be classified at all (even to kingdom) and 26 were classified only at the kingdom level to bacteria. The distribution of phyla, families and genera across the groups is shown in Figure 1 and Figure S1.

There were two dominant classes of bacteria, namely *Clostridia* and *Bacilli*. Bacteria from the class *Clostridia* mainly belong to two orders, which are *Lachnospirales* (all from one family, *Lachnospiraceae*) and *Oscillospirales* (which includes the *Ruminococcaceae* and *Oscillospiraceae* families). In contrast, bacteria of the class *Bacilli* belong to one of three orders, namely *Lactobacillales*, *Erysipelotrichales*, and *Staphylococcales*. All bacteria from the *Staphylococcales* group were from the *Streptococcaceae* family, while the *Lactobacillales* bacteria were classified into four families, namely *Lactobacillaceae*, *Streptococcaceae*, *Enterococcaceae*, and *Aerococcaceae*. The *Erysipelotrichales* order includes species from two families, namely *Erysipelatoclostridiaceae* and *Erysipelotrichaceae* [Figure 1B,C, Tables S1 and S2]. The most abundant bacterial species in all groups were *Akkermansia muciniphila* (the only species in Verrucomicrobiota), *Paraclostridium bifermentans* (*Clostridium bifermentans*), *Clostridium disporicum*, *Muricaecibacterium torontonense*, the group of bacteria *[Eubacterium] coprostanoligenes* (genus and species unknown), *Erysipelatoclostridium ramosum*, and *Ruminococcus bromii* [Figure 1C, Table S1].

Out of all the detected ASVs, only 494 were assigned at least to the phylum level [Table 1]. A total of 402 ASVs were present in at least 50% of the groups, and the number of ASVs in the samples varied significantly [Table 2]. Among these, 255 ASVs were detected in all groups, while the remaining ASVs were absent in at least one group. For instance, 16 ASVs were present in all groups except for group C3 (e.g., *Sellimonas intestinalis*, *Blautia faecis*, and *Citrobacter freundii*), and another 16 ASVs were detected in all groups except for group A3 (e.g., *Roseburia intestinalis*, *Roseburia faecis*, *Kineothrix alysoides*, and *Corynebacterium urealyticum*) [Figure S2, Table S3]. Only 196 ASVs were identified in all groups in most samples [Table 1].

The core microbiome (bacteria with significant abundance across the largest number of samples) comprised 53 ASVs. There were 26 of them in group A0, 32 in A1, 32 in A3, 36 in C0, 38 in C1, and 37 in C3. Only 16 of ASVs were found in all groups, including *Akkermansia muciniphila*, *Paraclostridium bifermentans*, *[Eubacterium] coprostanoligenes*, *Erysipelatoclostridium ramosum*, and *Ruminococcus bromii* [Figure S2B].

To further characterize ASVs of interest (those differentiating groups, the most abundant ASVs, those absent in a specific group, or the potential keystone species), the appropriate FASTA sequences were analyzed using BLAST (https://blast.ncbi.nlm.nih.gov/, accessed 24 September 2024) [19] to assign bacterial species where possible.

Since each ASV is assumed to represent a distinct bacteria species, some genera were represented by multiple ASVs, potentially indicating separate species. For example, the following genera had more than five distinct ASVs: *Erysipelatoclostridium* (18 ASVs), *Blautia* (12 ASVs), UCG-005 (*Oscillospiraceae*) (10 ASVs), *Allobaculum* (nine ASVs), *Colidextribacter* (nine ASVs), *Lachnospiraceae* NK4A136 group (seven ASVs), *Akkermansia* (six ASVs), *Corynebacterium* (six ASVs), and *Enterococcus* (six). After BLAST analysis, some of those ASVs were assigned to the distinctive species, as shown in Table S1.

**Figure 2:**
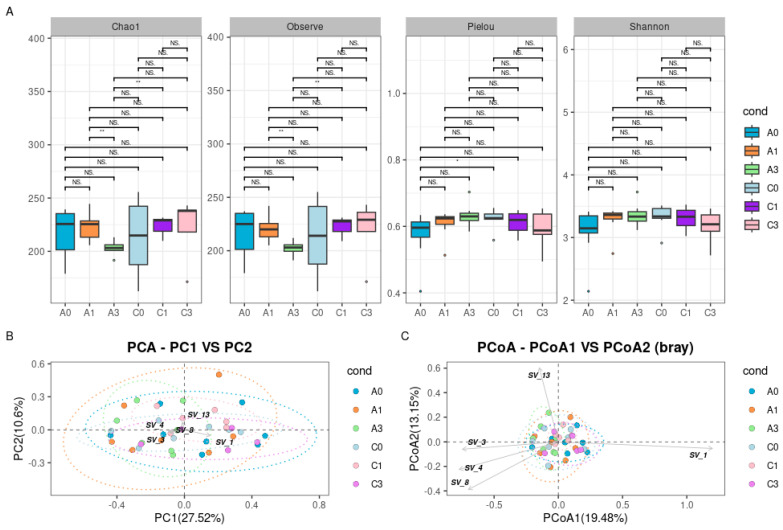
Colorectum bacterial profiles of control and CRC model groups including the diet regime information. Data from 6 groups (3 control and 3 CRC model) are shown. (**A**) Global community structure, bacterial diversity, evenness, and richness represented by observed species richness, the Chao1 index, Pielou’s evenness index, and Shannon diversity. Data are presented as box and whisker plots. Correction for multiple comparisons was made using the Benjamini–Hochberge procedure (BH; threshold of 0.05); * adjusted *p* < 0.05; ** *p* < 0.01; NS, not significant. (**B**) Hellinger-transformed species abundance data visualized with a principal component analysis (PCA) plot. The first two principal coordinate axes explain 27.52% and 10.6% of the variation, respectively. (**C**) Beta diversity assessed with Bray–Curtis visualized with a principal coordinate analysis (PCoA) plot. The first two principal coordinate axes explain 19.48% and 13.15% of the variation, respectively. ASV components with the highest significance are SV_1 *Akkermansia muciniphila*, SV_3 *Clostridium disporicum*, SV_4 *Leptogranulimonas caecicola*, SV_8 *Allobaculum* sp., and SV_13 *Blautia pseudococcoides.* Group description as in Figure 1.

The analysis of microbiota alpha diversity revealed that the species richness (Chao1 and observed) was lowest in the A3 group, with statistically significant differences (*p*-value < 0.05) when compared to groups A1 and C1. Although Shannon diversity did not show statistically significant differences in evenness, Pielou’s evenness index indicated statistically significant differences between groups A0 and C0 [Figure 2A].

The computation of beta diversity did not reveal distinct groups in either PCA and PCoA plots. *Akkermansia muciniphila* presented the most positive contribution to PC1 and PCoA1, while other ASVs, namely *Clostridium disporicum*, *Leptogranulimonas caecicola*, *Allobaculum* sp., and *Blautia pseudococcoides*, were negatively weighted. Of these, *Blautia pseudococcoides* was the only ASV contributing positively to PC2 and PCoA2 [Figure 2B,C].

**Figure 3:**
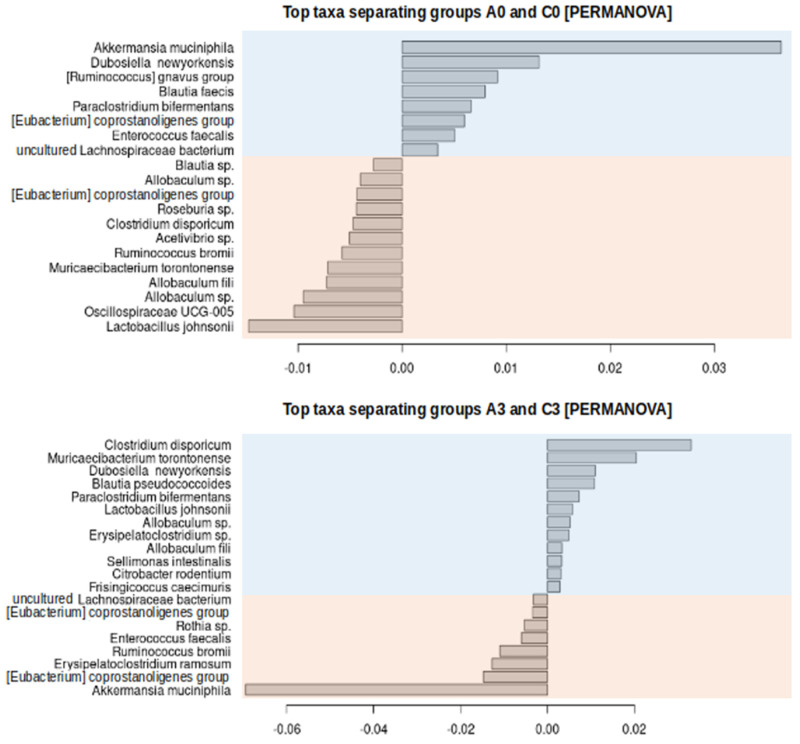
Dissimilarity in community composition between samples quantified by distance or divergence (PERMANOVA analysis results). Discriminating species of groups A0 and C0 (top) and A3 and C3 (bottom). The species with distances with values (*x*-axis) below 0 (orange background) are enriched in control groups, and those with values above 0 (blue background) are enriched in CRC groups. Group description as in Figure 1.

Beta diversity measures dissimilarity in the community composition between samples, and it can be quantified using the distance to the centroid or divergence measurements. These measures are widely used in statistical data analysis, for instance in a PERMANOVA. In our study, the PERMANOVA analysis did not show significant differences between the CRC and control groups. However, some not statistically significant trends are shown in Figure S3A,B, where the smallest divergence refers to A3 group samples. Despite the lack of significance in overall comparisons, paired comparisons between A0 and C0, as well as A3 and C3, revealed statistically significant differences (unadjusted *p*-values of 0.046 and 0.049, respectively). These differences also led to the identification of some discriminating species [Figure 3]. Notably, bacteria communities in the order *Clostridiales* in group A0 (the group with the highest number of ASVs in this order) differed significantly (*p*-value < 0.05, without correction for multiple repetitions) from all other groups. After introducing the B-H correction, group A0 remained significantly different only from groups A3 and C1.

An additional statistical analysis (pair comparison by Deseq2, ANCOMBC, Lefse, and Limma) identified several species that differentiated the investigated groups. However, none of them achieved an adjusted p-value below 0.05. Only those specimens which varied at least twice (LogFC < −1 or LogFC > 1) with a *p*-value < 0.05 (unadjusted p-value) in at least two of the statistical analyses are shown in Supplementary Materials Figure S3.

When the population size of bacteria families was calculated as the percentage of total bacteria abundance, some repeating patterns emerged among the most numerous bacterial families, independent of the sample type and dietary regimen. A statistically significant positive correlation was observed between *Erysipelotrichaceae* and *Atopobiaceae*, while these families negatively correlated with *Akkermansiaceae* [Figure S4, Table S2]. In groups supplemented with dietary fibres, the level of *Akkermansia muciniphila* decreased and the number of bacteria of the *Atopobiaceae* and *Erysipelotrichaceae* families increased [Figures S5 and S6]. In group A0, both *Akkermansiaceae* and *Lachnospiraceae* were elevated, while the level of *Erysypielotrichaceae* was reduced [Figure S7]. In group A0, the level of *Lactobacillus* was lower than that of C0 (*p*-value without adjustment = 0.0287). After oat beta-glucan supplementation, *Lactobacillus* level increased but not statistically significantly. The addition of 1% OBG increased the number of bacteria associated with butyrate production (*Lachnospiraceae* and *Ruminococcaceae*) compared to non-supplemented groups. Supplementation at the level of 3% OBG decreased the abundance of these families compared to the control without supplementation. This change was particularly evident in the C3 group, where bacterial diversity and the proportion of beneficial butyrate-producing bacteria decreased. In the C3 group, *Akkermansia muciniphila* levels increased to levels like those observed in the A0 group. Additionally, in the C3 group, the number of bacteria of the genus *Bifidobacterium* decreased, while the level of *Enterococcus faecalis* increased [Figure S8].

Microbial co-occurrence network analysis (with SPIEC-EASI—Estimation for Ecological Association Inference bioinformatics tool) was used to infer co-occurrence networks from 16S rRNA sequencing data. These methods infer ecological associations and can help understand species interactions, as microbes may engage with each other and interact with their environment (e.g., their hosts). Additionally, some species of bacteria of key importance (the keystone species) in a given environment (carrying unique functions that are essential for the balance of the microbiota) can be suggested, especially those creating multiple connections with other bacteria [20,21]. The analysis was performed on the 100 most abundant ASVs, which revealed two large ASV clusters (combining 48 ASVs and 15 ASVs) and eight smaller associations between several ASVs (from two to four ASVs) combining ASVs with very similar sequences of the analyzed 16s rRNA gene region. The remaining ASVs did not form any associations (16 ASVs) [Figure S9].

The smaller generated cluster contained mainly bacteria from the genus *Allobaculum* and *Erysipelatoclostridium*, while the bacteria gathered in larger one were from the families *Muribaculaceae*, and *Lachnospiraceae*, mostly from *Blautia* specie. Five ASVs formed several connections with other ASVs (from five to seven): *Blautia pseudococcoides* (seven), *Rothia* sp. (six), *Oscillibacter* sp. (six), *Muricomes intestini* (five), and an unassigned genus from the *Lachnospiraceae* family (five). *Blautia pseudococcoides* interacted directly with *Blautia faecis*, other *Blautia* sp., an unassigned genus from the *Anaerovoracaceae* family, the *Lachnospiraceae* family, *Eisenbergiella* sp., *Paramuribaculum intestinale*, and *Muricomes intestini*, all of which also formed multiple connections. *Muricomes intestini* seemed to interact with *Shuttleworthia* sp., *Blautia faecis*, *Blautia pseudococcoides*, *Clostridia UCG-014*, and *Anaerobutyricum soehngenii*. *Rothia* sp. interacted with *Akkermansia muciniphila*, *Ruminococcus bromii*, *Allobaculum fili*, *Streptococcus* sp., *Corynebacterium amycolatum*, and *Thomasclavelia ramosa*. *Oscillibacter* sp. formed connections with *Parabacteroides goldsteinii*, *Clostridia UCG-014*, the *Lachnospiraceae* NK4A136 group bacterium, *Anaerobutyricum soehngenii*, *Colidextribacter* sp., and *Oscillospiraceae* UCG-002. Finally, the *Lachnospiraceae* bacterium interacted with uncultured *Clostridium* sp., two uncultured *Muribaculaceae* bacteria, *Sellimonas intestinalis*, and *Citrobacter rodentium*.

### 2.3. Short-Chain Fatty Acid Profile in Colorectal Content

**Figure 4:**
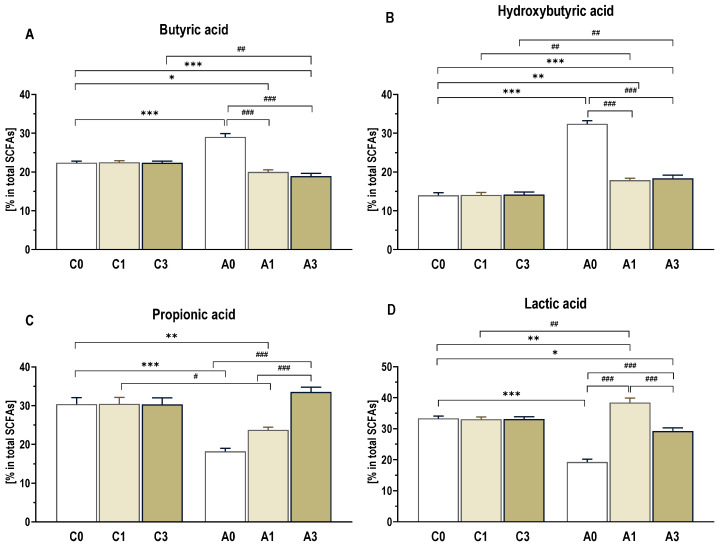
The SCFA profile in colorectal content. Changes in the profile of butyric acid (**A**), hydroxybutyric acid (**B**), propionic acid (**C**) and lactic acid (**D**) are presented as percentage of total SCFA content. Significant differences from the control group (C0) (* *p* < 0.05, ** *p* < 0.01; *** *p* < 0.001) (Tukey’s post hoc test). Significant differences within the control (C) and CRC (A) groups between dietary subgroups (# *p* < 0.05, ## *p* < 0.01, ### *p* < 0.001) (Tukey’s post hoc test). Group description as in Figure 1.

The colorectal contents of control rats showed the highest levels of lactic (33%) and propionic (30%) acids, with lower levels of butyric (20%) and hydroxybutyric (14%) acids [Figure 4]. There were no significant changes in these metabolites between the dietary subgroups within the control group. However, in the CRC rats, the profiles of these four colorectal microbiota metabolites differed significantly from those found in the control groups. In the A0 group, hydroxybutyric (32%) and butyric (29%) acids were the most abundant, while lactic (19%) and propionic (18%) acids were present at significantly lower levels. Dietary intervention with OBG, regardless of the dose, significantly reduced hydroxybutyric and butyric acid levels. In contrast, the propionic acid content increased proportionally with the dietary OBG supplementation. Additionally, lactic acid contents were higher in the A1 group than in the A3 group.

### 2.4. Lamina Propria and Intraepithelial Lymphocyte Profile

A cytometric analysis of the profile of immune cells isolated from the colonic walls of rats was performed for two populations of lymphocytes, intraepithelial lymphocytes (IELs) and lamina propria lymphocytes (LPLs). In both populations, total T lymphocytes, B lymphocytes, subpopulations of helper lymphocytes (Th), cytotoxic lymphocytes (Tc), and NK cells were distinguished.

**Figure 5:**
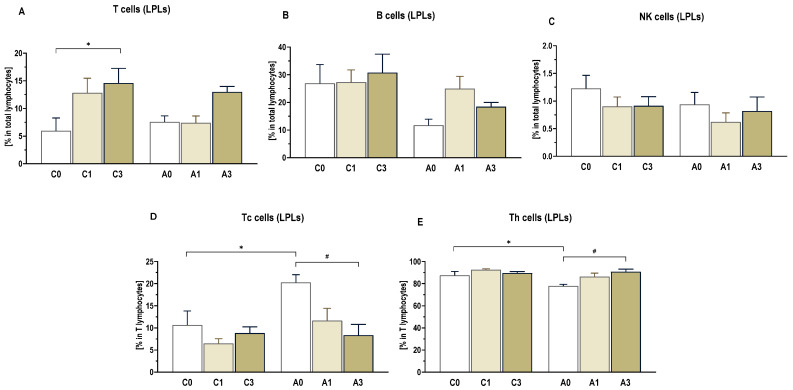
The profile of immune cells in colon lamina propria lymphocytes (LPLs). The profile of T (CD3+) cells (**A**), B (CD3−CD45RA+) cells (**B**) and natural killer (NK)(CD45+CD161a+) cells (**C**) are presented as percentage in total lymphocytes, while the profile of Tc (CD3+CD4−CD8+) cells (**D**) and Th (CD3+CD4+CD8−) cells (**E**) are presented as percentage in T lymphocytes. Significant differences from the control group (C0) (* *p* < 0.05) (Tukey’s post hoc test). Significant differences within the control (C) and CRC (A) groups between dietary subgroups (# *p* < 0.05) (Tukey’s post hoc test). Group description as in Figure 1.

In the colon population of LPLs [Figure 5], the percentage of T lymphocytes (CD3+) in CRC rats did not differ among the dietary groups (A0, A1, A3), whereas in the control groups, it was significantly higher (*p* < 0.05) in animals fed a diet supplemented with 3% OBG (C3) than in animals fed a diet without this supplement (C0). The percentage of Tc lymphocytes (CD3+CD4-CD8+) was higher in the CRC rats compared to the controls (A vs. C) (ANOVA, *p* < 0.02), with the highest value found in the A0 group fed the diet without OBG compared to the groups fed the diet with this additive, regardless of its level (A1 and A3) (*p* < 0.05). The percentage of Th lymphocytes (CD3+CD4+CD8-) was lower in CRC rats compared to controls (A vs. C) (ANOVA, *p* < 0.02), with the lowest value found in the A0 group fed the diet without OBG (*p* < 0.05). The percentage of B lymphocytes (CD3-CD45RA+) was lower in CRC rats (ANOVA, *p* < 0.05). There were no differences between CRC and control rats (A vs. C) based on the type of diet consumed. There were no differences in LPL NK cells (CD45+CD161a+) as a result of early-stage cancer development or diet supplementation.

**Figure 6:**
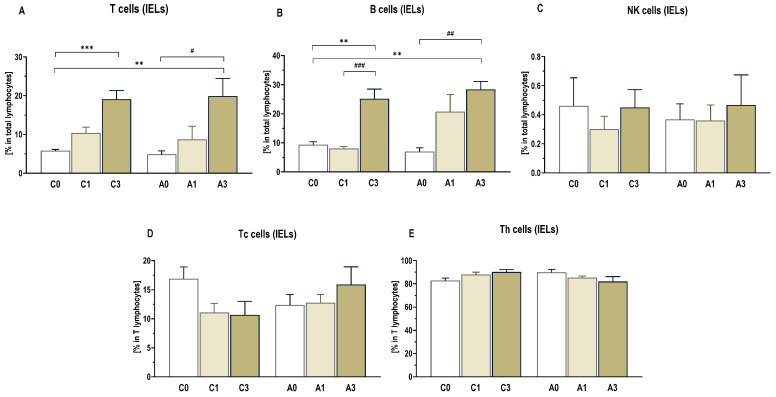
The profile of colon intraepithelial immune cells (IELs). The profile of T (CD3+) cells (**A**), B (CD3−CD45RA+) cells (**B**) and natural killer (NK)(CD45+CD161a+) cells (**C**) are presented as percentage in total lymphocytes, while the profile of Tc (CD3+CD4−CD8+) cells (**D**) and Th (CD3+CD4+CD8−) cells (**E**) are presented as percentage in T lymphocytes. Significant differences from the control group (C0) (** *p* < 0.01; *** *p* < 0.001) (Tukey’s post hoc test). Significant differences within the control (C) and CRC (A) groups between dietary subgroups (# *p* < 0.05, ## *p* < 0.01, ### *p* < 0.001) (Tukey’s post hoc test). Group description as in Figure 1.

The percentage of the intraepithelial T lymphocytes (CD3+) isolated from the colon tissue [Figure 6] was significantly higher in rats from groups C3 and A3 fed diets containing 3%OBG than in groups fed diets without this additive (C0 and A0) (*p* < 0.0 1). Similar differences were found for B lymphocytes (CD3-CD45RA+). There was a higher percentage of these cells in rats from groups C3 and A3 consuming diet with 3%OBG (*p* < 0.01). The percentages of Th (CD3+CD4+CD8-) and Tc lymphocytes (CD3+CD4−CD8+) and NK cells (CD45+CD161a+) in the population of IELs did not differ among the specific groups of animals.

### 2.5. Markers of Intestinal Barrier Integrity

**Figure 7:**
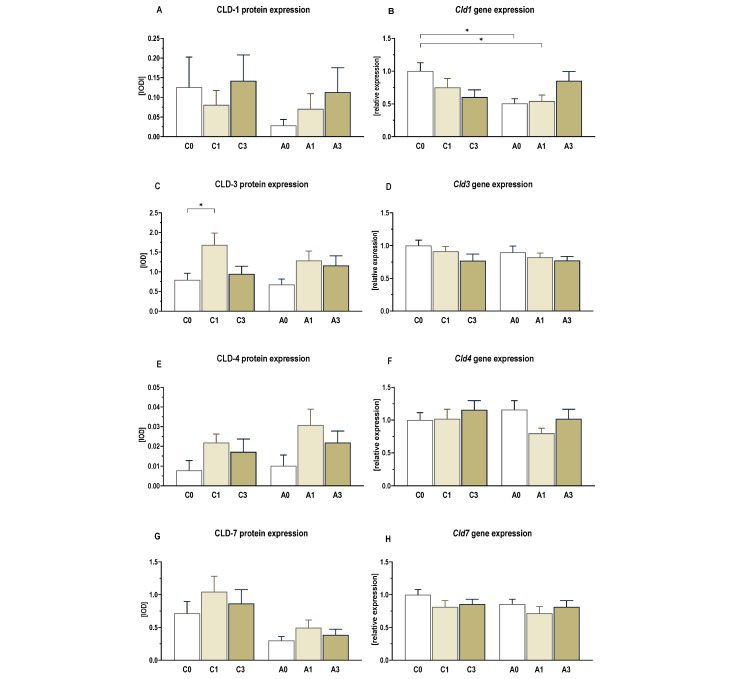

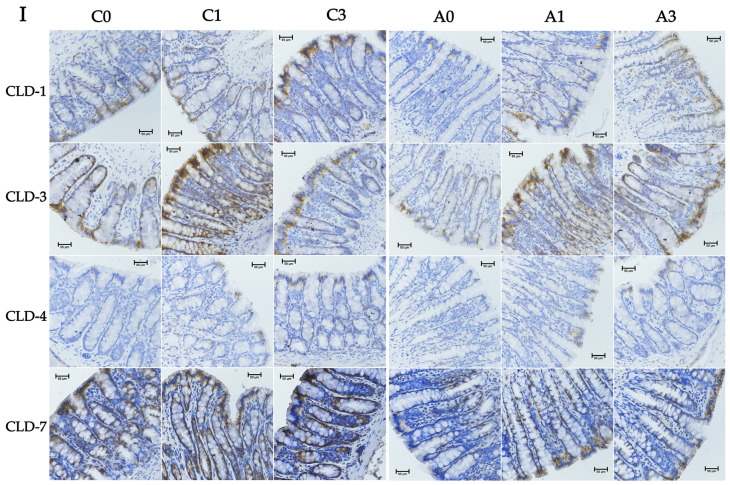
Gene and protein expression of claudins (CLD-1, 3, 4, 7) in the large intestine. Changes in the protein expression of CLD-1 (**A**), CLD-3 (**C**), CLD-4 (**E**), and CLD-7 (**G**) are presented as the integrated optical density (IOD) (mean ± SE). Light micrographs imaged (×400 magnification) (**I**). Brown precipitate indicates high expression of the analyzed claudins. Changes in the relative gene expression of *Cld1* (**B**), *Cld3* (**D**), *Cld4* (**F**), and *Cld7* (**H**) presented in arbitrary units as a ratio of the expression of the target gene to the mean expression of the reference genes (*B2m* and *Ldha*), with the control group calculated as 1. Significant differences from the control group (C0) (* *p* < 0.05) (Tukey’s post hoc test). Group description as in Figure 1.

The results of the immunohistochemical analysis of expression and localization of the intestinal barrier integrity proteins (claudin 1, 3, 4 and 7) are presented in Figure 7. The analysis of variance showed no significant interaction between the early stages of CRC development and dietary intervention on the expression of investigated proteins. However, the ANOVA indicated that the addition of OBG to the diet had an effect on the expression of claudin 3 and 4 (ANOVA, *p* < 0.01). Tukey’s post hoc analysis revealed a significantly higher expression of these proteins in the intestinal walls of rats fed a diet supplemented with 1% OBG (C1 group) compared to the group fed a diet without OBG (C0 group) (Tukey, *p* < 0.01). Furthermore, Dunett’s post hoc test showed a significantly higher expression of claudin 3 in the A1 group compared to the C0 group (control OBG-) (*p* < 0.05). In contrast, claudin 7 expression was affected only by the induced early stages of CRC, which resulted in a significant decrease in its expression (ANOVA, *p* < 0.001).

The studies also showed that in animals with CRC induced by AOM administration, the expression of genes encoding claudins (*Cldn3*, *Cldn4*, *Cldn7*), which play a key role in forming and regulating tight junctions essential for maintaining the epithelial barrier, remained unchanged (Figure 7). However, the ANOVA indicated that the gene expression of *Cldn1* was significantly affected by the health status of the animals, showing a downregulation in animals with CRC (ANOVA, *p* < 0.01). Tukey’s post hoc test confirmed a significantly lower mRNA expression of *Cldn1* in the intestinal walls of rats from the A0 and A1 groups compared to their respective control groups (Tukey, *p* < 0.05) [Figure 7B].

## 3. Discussion

In our 8-week experiment, we examined the effects of oat beta-glucan consumption on a rat model of CRC induced by azoxymethane. The induction of early-stage CRC caused significant alterations in the immune profile, intestinal barrier integrity markers, and microbiota composition. In rats with CRC, an increase in the percentage of Tc cells and a decrease in Th cells in the colon lamina propria were observed. The consumption of beta-glucan reversed these changes, especially when the feed supplement was added at a 3% concentration.

The results of the present study showed trends in gut microbiome changes as a result of AOM-induced CRC and the effect of OBG at different doses on both healthy and CRC rats. The results suggest a potential association between CRC development and an increased abundance of *Akermansiaceae*, *Lachnospiraceae*, and *Peptostreptococcaceae* families alongside a decrease in *Atopobiaceae* and *Lactobacillace*. Supplementation with OBG seemed to influence the abundance of *Erysypielotrichaceae* and *Atopobiaceae* families, increasing their level while simultaneously decreasing the level of *Akkermansiaceae*. While our study identified several trends suggesting potential effects, most changes did not reach statistical significance. This may be due to the small group sizes and the early stage of CRC used in the model, which could result in subtle changes that are inherently more difficult to detect statistically. In addition, the response of microorganisms to dietary fibres may vary between individuals, a phenomenon also observed in humans. The reasons for this variability are not fully understood but may be due to the absence of microorganisms important for fibre metabolism or the presence of strains lacking specific enzymes [22]. Nevertheless, the subtlety of these changes highlights the value of our findings, as they reveal early alterations in carcinogenesis that may become more pronounced at later stages.

Most bacteria detected in the large intestine of rats belong to the phyla *Bacteroidetes*, *Firmicutes*, *Actinobacteria*, and *Proteobacteria*, like the human large intestine microbiome. However, the ratio of these phyla varies depending on the experiments described [23,24]. A normal colon microbiota also contains facultative anaerobes, including *Lactobacilli*, *Enterococci*, *Streptococci*, and Enterobacteriaceae, present at levels about 1000 times lower. The exact number and variability of bacterial species depend on individuals [23]. In our experiment, the phylum *Firmicutes* dominated, followed by *Bacteroidetes* and *Verrucomicrobiota*. No optimal composition of the gut microbiome has been described so far. Differentiation is an individual trait related to factors such as enterotype, diet, or lifestyle [25]. Normally, in humans, about 40% of bacteria appear in all individuals, while the remaining 60% is variable and depends on many factors affecting the host organism [26]. So far, three human enterotypes have been described, characterized by distinguishing bacterial clusters and the different routes of obtaining energy in the fermentation process of substrates present in the large intestine. For example, enterotype one primarily derives energy from carbohydrates through glycolysis and the pentose phosphate pathway [25]. However, it is important to note that enterotypes represent loosely defined clusters of bacteria common within individuals rather than distinct traits, like blood type. Therefore, some researchers question their existence, especially since enterotypes can change under the influence of environmental conditions [27]. While no enterotypes have been described in rats, their presence cannot be ruled out, as they have been identified in other animals, such as primates or bumblebees, independent of age, origin, or gender [27]. In our experiment, we did not search for enterotypes, but we did notice some repeating patterns of the most numerous bacterial families that did not correlate with the sample type or dietary regimen, suggesting these patterns might be specific to the studied rats.

Our data show that *Akkermansiaceae* and *Erysypielotrichaceae* may compete. A similar interesting interaction between *Akkermansia* and *Allobaculum* (bacteria of the family *Erysypielotrichaceae*) was recently described by van Muijlwijk, who showed that *Allobaculum* secretes sialidase, targeting sialylated casein glycans critical for *Akkermansia muciniphila* growth [28]. An anti-correlation between the abundance of *Allobaculum* and *Akkermansia muciniphila* has been observed in both mice and humans [29]. Additionally, both species permanently co-colonized gnotobiotic mice and some humans. This suggests that the two species can coexist in selected environments [29]. A similar phenomenon was observed in our study.

Various diseases may cause alterations in the microbiota profile. Such changes have also been observed in CRC patients, including reduced diversity and altered composition [23,30,31]. The microbiome of CRC patients is typically enriched with *Proteobacteria* and opportunistic pathogens and depleted in butyrate-producing bacteria like *Lachnospiraceae* and *Ruminococcus*. *Bifidobacteriota* levels also decreased [25,32]. In CRC rats, a reduction in probiotic species like *Ruminococcus* and *Lactobacillus* and increases in potential pathogens have also been reported by other researchers [31,33]. In the present study, we observed only slight differences in the abundance of *Ruminococcus* bacteria from the *gnavus* group between the control group and the group with CRC, with a higher mean *Ruminococcus* abundance in the latter. Still, those differences did not reach statistical significance. In our study, we did not detect significant differences in the microbiota of rats with AOM-induced CRC, probably because the observed changes in our experiment are at a very early stage of CRC development. However, we observed elevated levels of *Akkermansia* and *Lachnospiraceae* in group A0, alongside a reduction in *Erysypielotrichaceae*. The families *Lachnospiraceae* and *Erysypielotrichaceae* and their role in disease processes are described ambiguously in the literature. For instance, *Ruminococcus gnavus (Lachnospiraceae)* is linked to Crohn’s disease and other gastrointestinal disorders, as it is the primary mucolytic bacterium in Crohn’s disease. In contrast, the levels of other bacteria in this family are reduced [34,35]. Other species of *Lachnospiraceae*, e.g., *Blautia* and *Roseburia*, are involved in controlling intestinal inflammation and immune system maturation through SCFA production. Thus, they have a beneficial effect on the health of the host [34]. Conversely, *Allobaculum* (*Erysypielotrichaceae*) has been associated with enhanced intestinal inflammation and lipid metabolism, particularly in high-fat diets, potentially contributing to obesity [36].

The results of our experiment showed that the abundance of *Akkermansiaceae* was high in most cases, with *Akkermansia muciniphila* being the most abundant species. *Akkermansia* was originally thought to be a species of bacteria found in the human digestive system, and its presence was rarely detected in rodents [27,37]. However, it is now considered a key species present in the physiological microbiota of the rodent digestive system, playing a crucial role in maintaining gut function, similar to its role in humans [38]. While some individuals in our study exhibited very low levels of *Akkermansia muciniphila*, it was the most abundant bacterial species in most cases. An increased abundance of *Akkermansia muciniphila* in the literature is most often correlated with a normal body condition, while a decrease correlates with a disease state [39]. A significant reduction in the presence of *Akkermansia muciniphila* may impair intestinal barrier function and promote inflammation [38]. On one side, *Akkermansia* is considered a key species that regulates the immune response, improves intestinal barrier integrity, and is associated with protection against CRC [39,40]. However, studies on the high-dose supplementation of *Akkermansia muciniphila* have shown adverse effects, especially after antibiotic therapy. In such cases, *Akkermansia* disrupted the intestinal barrier, caused dysbiosis, exacerbated inflammation, and contributed to cancer development [41]. The results of our study indicate a complex relationship between the gut microbiota, particularly *Akkermansia muciniphila*, and the regulation of intestinal barrier integrity in CRC.

We also found that induced CRC development resulted in a reduction in only CLD-7 protein expression and *Cld1* gene expression due to the early stage of carcinogenesis. OBG consumption did not affect CLD-7 expression but affected *Cld1* gene expression levels, increasing values in the CRC group consuming a diet with 3% OBG. In addition, the consumption of OBG, particularly at the level of 1%, increased the expression of CLD-3 and CLD-4 proteins independently of the induced CRC. Reduced expression of CLD-3 and CLD-7 proteins was also observed in human colon cancer, including the early stages of cancer development, which may accelerate epithelial barrier damage [42,43,44,45]. Several studies indicate that the expression of some claudins, including claudin-1, 3, and 7, was increased in CRC. However, increased expression of these proteins in tumour tissue is a favourable prognostic indicator, associated with better overall survival, reduced risk of metastasis, and negative venous and lymphatic invasion. This is supported by many studies, which are cited in a meta-analysis of CLD-1 results and in a review article [46,47]. To summarize the above, our results suggest that OBG consumption enhances intestinal barrier integrity by increasing the expression of several barrier proteins, while the development of CRC can decrease the expression of other key barrier proteins. Moreover, these findings suggest that while *Akkermansia muciniphila* plays a crucial role in maintaining gut health, its dysregulation, along with compromised barrier proteins like CLD-1 and CLD-7, may contribute to CRC progression, which dietary OBG can partially counteract through the upregulation of CLD-3 and CLD-4.

In addition, an increased abundance of bacteria from the *Verrucomicrobiota* family, which includes *Akkermansia muciniphila*, has been linked to reduced levels of *Actinobacteria* [38]. A similar relationship was observed in our experiment, where a high abundance of *Akkermansia* was often associated with low or undetectable levels of *Actinobacteria*. *Atopobiaceae* (*Actinobacteria*) are considered health-beneficial bacteria, especially for preventing colonization by harmful bacteria in elderly individuals [48]. Our experiment showed a statistically significant correlation between *Erysipelotrichaceae* and *Atopobiaceae*, with a negative correlation between these families and *Akkermansiaceae*. This suggests that a reduced level of *Akkermansiaceae* in relation to *Erysipelotrichaceae* may not always be a pathological symptom, especially since supplementation with dietary fibres led to a decrease in the level of *Akkermansia muciniphila* and an increase in the number of bacteria of the *Atopobiaceae* and *Erysipelotrichaceae* families.

Some taxa, such as *Akkermansia*, preferentially replicate during colitis development. It has been shown that the composition of the gut bacterial community in mice shifts toward faster-replicating taxa as gut inflammation progresses [49]. The dynamics of bacterial growth were likely important in the case of an increase in the number of *Akkermansia* in A0 rats. Similarly, elevated levels of *Akkermansia muciniphila* in CRC patients were demonstrated, which could be a consequence of overexpression of certain types of mucins in CRC. Mucus prevents the development of CRC, but in the late stages of cancer, it contributes to tumour growth and disease progression [50]. *Akkermansia* was positively correlated with CRC growth and size, with its abundance significantly increased in CRC patients and mouse models of CRC [31]. In our experiment, we observed the highest levels of *Akkermansia muciniphila* in groups A0 and C3, but the reasons for the increase in this bacterium are most likely different. A diet devoid of dietary fibre increases the expression of carbohydrate-active enzymes (CAZymes) of the gut microbiota that target the degradation of mucins and promotes the expansion of mucin-degrading bacteria, such as *Akkermansia muciniphila* [51]. In the C3 group, the increase in *Akkermansia muciniphila* may be related to its ability to metabolize glucose and other sugars, potentially provided by the high levels of OBG in the diet, which serve as an easily available source of energy for these bacteria [39].

The microbiota’s fermentation of OBG in the cecum and colon supports the growth of beneficial bacteria, such as *Lactobacillus*, *Bifidobacterium*, and *Akkermansia*. SCFAs produced in the OBG fermentation can inhibit CRC initiation [52]. Specialized bacteria originally involved in the breakdown of OBG provide substrates for butyrate producers that cannot directly metabolize dietary fibres [30,53]. However, the microbial response to dietary fibre is highly individualized and not fully understood. Some individuals may lack species capable of degrading specific dietary fibres or strains with the enzymatic capacity to utilize these substrates [22]. Therefore, the host’s response is context-dependent on the surrounding microbial community, as with differential responses to pathogens [29].

In our study, the effects of β-glucan consumption had varying effects. We observed an increase in the number of beneficial species, such as *Paraclostridium bifermentans*, *Romboutsia* sp., and *Lactobacillus* after the administration of OBG in rats with CRC, although the control group showed a smaller effect. For example, *Lactobacillus* levels in group A0 were lower than C0 (*p*-value without adjustment = 0.0287). After OBG consumption, the *Lactobacillus* level increased but not statistically significantly. Supplementation with 3% OBG caused noteworthy changes in gut microbiota composition in the control group. In the C3 group, we observed a decrease in bacterial diversity and a percentage decrease in the number of beneficial bacteria, especially those producing butyrate. On the other hand, *Akkermansia muciniphila* levels increased significantly (to a level close to the average level in the A0 group). *Akkermansia muciniphila* uses mucins as the main source of carbon and nitrogen, but in the absence of mucins in the environment, it can switch to glucose metabolism via glycolysis [41]. This ability might explain its growth in the C3 group, which had access to easily metabolizable glucose derived from the breakdown of high-dose OBG. The increase in the number of *Akkermansiaceae* was not observed in group A3, suggesting potential differences in the microbial species that depend on the health status of rats.

We also found that adding 1% OBG increased the number of bacteria associated with butyrate production, such as *Lachnospiraceae* and *Ruminococcaceae*, compared to the non-supplemented groups. In contrast, 3% OBG supplementation decreased their abundance compared to the control without supplementation, which is particularly evident in the C3 group. In the C3 group, the number of bacteria of the genus *Bifidobacterium*, i.e., bacteria with probiotic activity, also decreased, and the level of the potentially harmful bacterium *Enterococcus faecalis* increased. *Enterococcus faecalis* is typically considered a commensal organism; however, recent studies have implicated its involvement in the development of CRC [54]. It has been observed that *Enterococcus faecalis* is more prevalent in CRC patients, as this bacterium can form biofilms and produce collagenase, both of which may promote tumour growth, invasion, and migration [55,56]. Additionally, *Enterococcus faecalis* activates can stimulate angiogenesis by inducing VEGFA and interleucin-8 [57]. On the other hand, some studies suggest a probiotic role of this bacterium in preventing CRC, potentially due to its ability to produce butyrate, a short-chain fatty acid with anti-inflammatory properties [58]. However, direct evidence linking *Enterococcus faecalis* to cancer development is lacking, and other researchers have found no association between this bacterium and CRC [26,59]. Such conflicting results may be explained by variability among different strains of *Enterococcus faecalis*, that have been isolated and studied. Through gene transfer, these bacteria can become more or less virulent. Moreover, our findings have shown only an increase in its concentration without clarifying its functional role in CRC development. It is possible that *Enterococcus faecalis* acts merely as a “passenger” bacterium, becoming pathogenic only when drastic environmental changes occur—such as those seen at the onset and progression of CRC—including shifts in cytokine and mucin production or alterations in oxygen tension [55]. Additionally, the butyrate production in the C3 group did not change significantly, likely because the decline in the number of bacteria from the *Lachnospiracea* and *Ruminococcaceae* families was relatively small and not statistically significant. On the other hand, in group A3, butyrate production might have increased due to the higher abundance of *Clostridia*, which are known as butyrate producers. The observed increase in the percentage of butyric acid in group A0 in the present study may have resulted either from a reduction in the synthesis of other fatty acids or from a significant increase in the abundance of *Akkermansia muciniphila*. As suggested by the study by Geerlings et al., *Akkermansia muciniphila* may stimulate butyrate synthesis, possibly by releasing substrates for butyrate producers from decomposed mucilage [39]. Interestingly, a comparison between CRC rats with and without 1% OBG supplementation showed an average decrease in the number of *Akkermansiaceae* and an increase in the number of *Erysypielotrichaceae*.

The search for keystone bacteria did not bring the expected results, as many identified bacteria shared similar metabolic functions or belonged to the same genus. Similar correlations between species from the same genus were observed in other studies on rats [60]. An interesting analysis of the microbiota structure of patients treated with antibiotics for a long time showed a strong disruption in the bacterial community structure. The depleted microbiota was characterized by many centres clustered by bacteria of the *Clostridia* class, while in untreated people, the centre was *Akkermansia muciniphila*. This suggests a substantial reconstruction of the network of dependencies between bacteria in the gut [61]. The co-occurrence network of bacteria obtained in our experiment is also fragmented with the dominant bacteria of the class *Clostridia*. Makki et al. [22] proposed that key bacterial species might be those specialized in breaking dietary fibres, such as *Ruminocccus bromii*, which is associated with resistant starch fermentation. However, our experiment did not allow us to determine the key bacterial species involved in OBG degradation, the existence of which was suggested by Makki et al. [22].

Our results indicate that the effect of oat beta-glucan is less noticeable in animals with a healthy colon, which is aligned with our earlier observations. Previous studies demonstrated that the anti-inflammatory effects of oat beta-glucan in the gastrointestinal tract were most notable in animals with LPS-induced enteritis as well as TNBS-induced colitis [13,15,62]. Thus, we suggest that oat beta-glucans may be more effective as an accessory therapy element during active disease conditions rather than as a preventive measure in healthy subjects. Moreover, these properties suggest that OBG consumption is fundamentally safe.

This study has several limitations that should be acknowledged. While trends in gut microbiota composition and intestinal barrier integrity were observed, many changes did not reach statistical significance. This is likely due to the relatively small sample size, which, while ethically necessary, limits the ability to detect subtle effects with confidence. Achieving statistical significance would require a larger group of animals, but this presents ethical challenges in line with animal welfare guidelines. Moreover, using an early-stage CRC model may have resulted in subtle changes that were difficult to detect at this stage of carcinogenesis, and these changes may become more pronounced at later stages. Additionally, as highlighted in the literature, variations in microbiota results are a common issue affecting all microbiota profile assessment studies due to technical factors such as sample storage, DNA isolation methods, and sequencing protocols. These factors can ultimately influence the results, making it difficult to compare our findings with those from other studies [63,64]. Moreover, without knowledge of the initial microbiome composition of individual rats, it is also challenging to determine whether observed changes are biologically relevant. On the other hand, in our study, we analyzed the microbiome composition of colonic contents collected post-mortem, providing a precise information about the microbiome at the site of interest. Unlike fecal samples, which can be collected non-invasively at multiple time points, colonic content sampling requires sacrificing the animal to obtain material directly from the colon lumen. We chose this approach because our primary aim was to compare microbiome profiles between different experimental groups at the study endpoint rather than assess changes over time within individual animals. By sourcing animals from Charles River Laboratories, randomly assigning them to groups, and housing them under identical conditions, we minimized inter-individual variability and assumed comparable baseline microbiome compositions across groups. The significant differences observed between groups suggest that the dietary intervention with oat beta-glucan and AOM treatment had a measurable impact on colonic microbiome composition. This study’s findings, while valuable, indicate the necessity of further investigation.

## 4. Materials and Methods

### 4.1. Low-Molar-Mass Oat Beta-Glucan Isolation and Characterization

A low-molar-mass oat 1-3, 1-4, beta-D-glucan isolate (OBG) was obtained from oat bran (Bestpharma, Warsaw, Poland) using a unique, patented extraction method as described in our previous studies [65,66]. The detailed methodology for the isolation and purification of beta-glucan is presented in our previous article [17]. Briefly, after several freeze–milling cycles of oat bran for particle size reduction, the beta-glucan alkaline extraction proceeded (pH 8.5, NaOH). The insoluble fraction was removed by centrifugation and the supernatant was subsequently deproteinized at the isoelectric point. The purified beta-glucan preparations showed a purity level of 99.3%, as confirmed by enzymatic analysis using the Megazyme Kit (Megazyme by Neogen, Bray, Ireland). The molar mass of the beta-glucan isolate was determined to be 52 ± 6 kDa by size exclusion high-performance liquid chromatography (HPLC), consistent with previously published data [65]. Additionally, protein content was measured using the Lowry method, with results indicating levels below 0.01 mg/mL.

### 4.2. In Vivo Experiment Procedures

**Figure 8:**
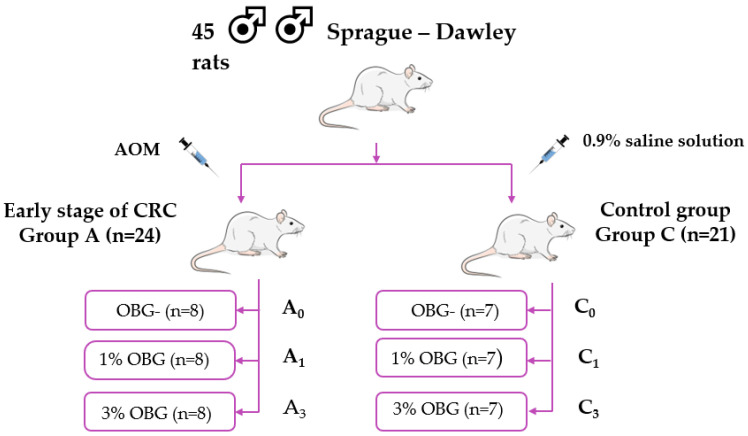
Scheme of the in vivo study. AOM—azoxymethane; CRC—colorectal cancer (AOM-induced early stage of colorectal carcinogenesis); 1% OBG—feed containing 1% (*w*/*w*) low-molar-mass oat beta-glucan; 3% OBG—feed containing 3% (*w*/*w*) low-molar-mass oat beta-glucan; OBG-—feed without low-molar-mass oat beta-glucan.

The experiment was performed on 45 eight-week-old male outbred Sprague Dawley rats Crl:CD (SD) (Strain Code 001, Charles River Laboratories, Sulzfeld, Germany), all specific pathogen-free (SPF) with an initial body weight of 272 ± 11 g. All rats were housed individually under stable environmental conditions, with a temperature of 23 °C, 60% relative humidity, 15 air changes per hour, and a 12:12 h light–dark photoperiod. After 10 days of acclimatization and handling, the rats were divided into 2 main groups, a group with chemically induced early-stage CRC (A, n = 24) and a control group with no pathological changes in the colorectum (C, n = 21). Early-stage CRC was induced by peritoneal injection of azoxymethane (AOM) (Sigma-Aldrich, Saint Louis, MO, USA). The AOM was administered twice, one week apart, at a dosage of 15 mg/kg each time. Control animals received saline in the same manner. After AOM/saline administration, both A and C groups were divided into 3 dietary subgroups, receiving either a standard AIN-93M diet (ZooLab, Sędziszów, Poland) without OBG (groups A0 and C0), an AIN-93M diet supplemented with 1% (*w*/*w*) OBG (groups A1 and C1), or an AIN-93M diet supplemented with 3% (*w*/*w*) OBG (groups A3 and C3). The scheme of animal experiments is presented in Figure 8. The sample sizes for each group were calculated based on the minimum needed for statistical power and were determined based on the scientific literature [67] using online available tools [68]. Feed was weighed at approximately 26 g per day per rat, and the measured average feed intake was 25.34 ± 0.25. The detailed methodology of this in vivo experiment is described in detail in our previous publication [69].

Body weight gain was recorded weekly, while food consumption and general health status were monitored daily. At the end of the 8-week feeding period, the rats were bled from the heart under deep isoflurane (Aerrane Isoflurane USP, Baxter, Poland) anesthesia. After bleeding, the three parts of the large intestine (cecum, colon, and rectum) along with their contents were collected. The intestinal contents were immediately frozen in liquid nitrogen and stored at −80 °C for subsequent DNA extraction. Parts of all large intestine sampled sections were thoroughly rinsed with PBS buffer and were allocated for cytometric analysis to assess immune nuclear cell subpopulations. Samples from each section of the large intestine were fixed in 10% buffered formalin for histological and histopathological evaluation, as described in detail previously [17].

All procedures were approved by the second Local Ethics Committee in Warsaw, Poland (resolution no. # WAW2/040/2019, 15 March 2019) and conducted in accordance with the EU Directive (2010/63/UE), Polish law, and the principles of the 3R rules.

### 4.3. RNA Extraction, cDNA Synthesis, and Analysis of Gene Expression

RNA was isolated from large intestine samples using the RNeasy Lipid Tissue Mini Kit (Qiagen, Hilden, Germany) according to the manufacturer’s instructions. The purity and concentration of the isolated RNA were verified using the NanoDrop™ 2000 spectrophotometer (Thermo Fisher Scientific, Waltham, MA, USA), while RNA integrity was evaluated on selected RNA samples using the Agilent Bioanalyzer 2100 system and RNA 6000 Nano LabChip^®^ Kit (Agilent Technologies, Palo Alto, CA, USA). This evaluation showed minimal RNA degradation, as demonstrated by an RNA integrity number (RIN) above 9. Complementary DNA was synthesized using the RT2 First Strand Kit (Qiagen, Hilden, Germany), and gene expression analysis focused on selected CRC-associated pathways was performed using the Custom RT2 Profiler™ PCR array (Qiagen) following the manufacturer’s instructions. To verify the results’ accuracy and reliability, each colon sample was analyzed in duplicate. The Custom RT2 Profiler PCR arrays (Qiagen, Hilden, Germany) incorporate primers designed for the detailed study of genes critical to gut barrier integrity, including *Cldn1* (catalogue number PPR47814B), *Cldn3* (catalogue number PPR43424A), *Cldn4* (catalogue number PPR43702B), and *Cldn7* (catalogue number PPR48513A). Amplification was carried out using the AriaMx Real-time PCR System (Agilent Technologies, Palo Alto, CA, USA), beginning with a 10 min denaturation at 95 °C, followed by 40 cycles of 15 s at 95 °C for denaturation and 1 min at 60 °C for annealing/extension. Relative gene expression levels were calculated using the ΔΔCt approach, with *B2m* (catalogue number PPR42607A) and *Ldha* (catalogue number PPR56603B) as reference genes for normalization [70]. The levels of target gene expression, normalized to *B2m* and *Ldha*, are presented as fold changes relative to the control group, which is set at a baseline value of 1 to illustrate gene expression changes.

### 4.4. DNA Extraction

Samples of frozen colon contents were stored at −80 °C before DNA extraction. Genomic DNA was extracted from the samples using the QIAamp PowerFecal ProDNA isolation kit (Qiagen, Hilden, Germany). In brief, approximately 200 mg of colon material was placed into PowerBead Pro tubes, to which 800 μL of CD1 solution was added and thoroughly mixed by vortexing. The samples were then processed using an Omni^®^ Bead Ruptor 12 Homogenizer (OMNI International, Kennesaw, GA, USA) with initial homogenization at a speed of 3.1 m/s for 30 s to improve cell lysis, followed by two steps of 30 s homogenization at a speed of 1 m/s with a periodic 5 min pause. After homogenization, the tubes were centrifuged at 15,000× *g* for 1 min. The clear supernatant was then carefully transferred to sterile microcentrifuge tubes, and subsequent steps of the extraction process were conducted according to the manufacturer’s instructions. DNA concentrations and purity (A260/A280 ratio) were measured using a NanoDrop™ 2000 spectrophotometer (Thermo Fisher Scientific, Waltham, MA, USA). The DNA samples were subjected to agarose gel electrophoresis to assess their quality. We confirmed high-molecular-weight DNA with no RNA contamination.

### 4.5. 16S rDNA Microbiome Sequencing

DNA isolated from intestinal content samples was used for library preparation and subsequent sequencing of the 16S rDNA amplicon (V3–V4 region) on the Illumina Miseq platform (Illumina, San Diego, CA, USA). The PCR primers, i.e., forward (TCGTCGGCAGCGTCAGATGTGTATAAGAGACAGCCTACGGGNGGCWGCAG) and reverse (GTCTCGTGGGCTCGGAGATGTGTATAAGAGACAGGACTACHVGGGTATCTAATCC), were designed to synthesize a single amplicon of approximately ~460 bp of the hypervariable regions (V3–V4) of 16S rRNAs. Library preparation was carried out according to Illumina protocol “16S Metagenomic Sequencing Library Preparation. Preparing 16S Ribosomal RNA Gene Amplicons for the Illumina MiSeq System”, increasing the number of cycles from 25 to 28 in the amplicon synthesis PCR reaction. The synthesized libraries were finally pooled and sequenced with a 10% phiX spike-in on an Illumina MiSeq sequencer using the paired-end 2 × 300 bp v3 reagent kit, according to the manufacturer’s instructions.

### 4.6. Isolation of Lymphocyte Subpopulations from Large Intestine Walls

Segments of large intestine tissue were cut into 2–3 mm fragments and incubated in flasks containing 2 mM dithiothreitol (DTT) in Ca^2+^- and Mg^2+^-free Hank’s balanced salt solution (HBSS) at 37 °C. Next, the suspension of detached cells and tissue fragments were filtered through cell strainers (70 µm pore size, BD Falcon, Franklin Lakes, NJ, USA). The collected filtrate containing isolated intraepithelial lymphocytes (IELs) was centrifuged at 400× *g* for 5 min at 4 °C. The supernatant was removed, and IELs were suspended in PBS with 2% fetal bovine serum (FBS) and left at 4 °C until further steps of the immunofluorescence staining protocol. The remaining colon tissue fragments on the strainers were suspended in RPMI 1640 medium containing 5% FBS, HEPES (5.96 g/L), 2.4 mg of collagenase type I (214 units/mg), and antibiotics (100 mg/L of streptomycin sulphate and 7 mg/L of penicillin G potassium) in order to release leukocytes from the *lamina propria* leukocytes (LPLs) of the large intestine tissue. Enzymatic digestion was carried out for 1 h at 37 °C with constant vigorous shaking (200 rpm). The digestion was terminated by an addition of ice-cold RPMI medium supplemented with 5% FBS and antibiotics when the suspension became transparent. Next, the cell suspension was filtered through cell strainers (40 µm pore size, BD Falcon, USA) and centrifuged at 400× *g* for 5 min at 4 °C. The pellets containing lamina propria lymphocytes (LPLs) were resuspended in PBS supplemented with 2% FBS and used for immunofluorescence staining.

### 4.7. Immunostaining and Cytometric Analysis of Lymphocyte Subpopulations Isolated from Large Intestine Walls

Aliquots of the cellular fractions (IELs and LPLs) suspended in 100 μL of PBS with 2% FBS were prepared for subsequent flow cytometric analysis.

Antibodies against the following surface markers were used: (1) APC anti-rat CD3 (marker of lymphocyte population); (2) FITC anti-rat CD45RA (marker of lymphocyte B population); (3) PE anti-rat CD161a (marker of NK population); (4) PE anti-rat CD4 (marker of lymphocyte Th subpopulation); (5) FITC anti-rat CD8a (marker of lymphocyte Tc subpopulation); and (6) APC-Cy7 anti-rat CD45 (leukocyte common antigen). These antibodies were provided by BD Pharmingen (San Diego, CA, USA) rat-specific cocktails, namely a rat T/B/NK antibody cocktail (catalogue number: 558495) and rat T-cell cocktail (cat no. 558493). Immunofluorescent staining was performed following the protocols recommended by the manufacturer of the antibody cocktails.

An analysis of different lymphocyte subpopulation profiles was performed using the flow cytometer FACSAria II (BD Biosciences, Franklin Lakes, NJ, USA). The lymphocyte population was first gated based on morphological characteristics, such as forward scatter (FSC) and side scatter (SSC) (gate P1). Cells located in gate P1 were then analyzed with regard to their positive staining with appropriate antibodies. Data were collected from at least 10,000 lymphocytes in gate P1. Unstained cells were used as a negative control. The compensation procedure was performed using a rat compensation set provided by BD Pharmingen.

### 4.8. Immunohistochemical Analysis

The immunohistochemical analysis was performed as described by Kopiasz et al. [69]. Briefly, tissue slices (5 µm thickness) containing three representative pieces of the large intestine from each rat were used. These tissues were deparaffinized in xylene and rehydrated in a series of decreasing concentrations of ethanol. After blocking endogenous enzymes and recovering antigens, samples were incubated for overnight at 4 °C with primary antibodies—a mouse anti-Claudin 1 monoclonal antibody (Cat#37-4900, 1:200), rabbit anti-Claudin 3 polyclonal antibody (Cat#PA1-37469, 1:250), mouse anti-Claudin 4 monoclonal antibody (Cat#32-9400, 1:200), and a rabbit anti-Claudin 3 polyclonal antibody (Cat#PA5-32356, 1:800). All antibodies were from Invitrogen, Thermo Fisher Scientific, Waltham, MA, USA. The following day, the samples were labelled with polymers consisting of anti-rabbit or anti-mouse antibodies conjugated to the horseradish peroxidase (HRP) enzyme complex. 3,3′-Diaminobenzidine (DAB) was used for brown staining, and haematoxylin was utilized for nuclei counterstaining. The immunohistochemically stained slides were examined using a NIKON Eclipse Ti2 microscope (Nikon, Melville, NY, USA). On the recorded images, six mucosal areas for each of the three sections of the colorectum were marked. A total of eighteen marked mucosal areas for the colorectum from one rat were analyzed collectively. Colourimetric saturation (brown colours reflecting antigen expression) and object area were quantified using the NIS-Elements BR 5.01 programme.

### 4.9. SCFA Level Analysis

The profile and concentration of SCFAs in the colon content samples were determined using HPLC with UV detection. The mobile phase for the isocratic elution of SCFAs was composed of 15 mM monobasic sodium phosphate/methanol (80:20). Analyses of the level of propionic, butyric, hydroxybutiric acetic, and lactic acid using pure standards using a liquid chromatography system with UV detection [HPLC-DIONEX P680 (Dionex, Sunnyvale, CA, USA)] with an autosampler [Dionex ASI-100, Dionex, USA] were performed. The concentration of the SCFAs was expressed in relative values, with the sum of all the acids determined being taken as 100%.

### 4.10. Data Analysis and Visualization

All microbiome profile analyses were conducted using R 4.3.0 [71] in RStudio 2023.06.1+524. The quality of the raw paired-end reads was evaluated before the analysis using the FastQC tool (“fastqcr” v 0.1.3.999) [72]. Obtained sequences were processed using DADA2 (“dada2” v 1.30.0) [73], including sequence trimming, quality-based filtering, sequence inferring, merging, and chimera removal, resulting in 1024 ASVs (amplified sequence variants). After excluding low-abundance reads (<0.0001%) and singleton ASVs using “phyloseq” v1.46.0 [74] and “genefilter” v 1.84.0 [75] packages, the remaining 532 ASVs were compared to the SILVA v 138.1 database (https://ngs.arb-silva.de/silvangs/, accessed 12 August 2023). This comparison allowed for classification into a microbial taxonomy at various levels (phylum, class, order, family, genus). Approximately 56% of the total sequences were successfully classified (median per sample 154,365; IQR 142,531.2–170,826.8). The ASV table was further processed using the R packages “phyloseq” v 1.46.0 and dplyr v 1.1.4 [76]. Plots were generated using the R package “ggplot2” v 3.4.4 [77] and “patchwork” v 1.1.3 [78]. Alpha diversity Chao1, Shannon, Pielou indexes, and the Wilcox test were calculated using the “MicrobiotaProcess” v 1.14.0 [79].

A two-way permutation multivariate analysis of variance (“Two-way PERMANOVA”) was conducted using the R package “vegan” v 2.6.4 [80] to test the differences in microbial composition between groups. β-diversity (species complexity) was estimated by principal component analysis (PCA) and principal coordinate analysis (PCoA) based on the Bray–Curtis distance using the relative values of ASVs at the genus level with the same package. Differential abundance testing was performed using the “microbiomeMarker” v. 1.8.0 [81] applying multiple statistical approaches, such as DESeq2 (based on the negative binomial distribution), limma voom (mean variance modelling at the observational level), and the linear discriminant analysis (LDA) effect size (LEfSe) algorithm. Additionally, the analysis of the composition of microbiomes with bias correction (ANCOM-BC) was conducted using the “ANCOMBC” v. 2.4.0.

The Benjamani–Hochberg’s correction (with adjusted *p*-values < 0.05) was used to correct for multiple comparisons. To identify microbial groups and potential microbe-to-microbe interactions, the SPIEC-EASI (SParse InversE Covariance Estimation for Ecological ASsociation Inference, pronounced speakeasy) [82] statistical method was used (R package “SpiecEasi” v. 1.1.3). A further characterization of ASVs of interest was achieved by analyzing the appropriate fasta sequences with BLAST (https://blast.ncbi.nlm.nih.gov/, accessed 24 September 2024) to assign bacterial species when possible.

The data obtained for the analysis of short-chain fatty acid profiles, lymphocyte profiles, and markers of intestinal barrier integrity were analyzed using Statistica software (version 13.3 PL; StatSoft, Cracow, Poland). The normality of distribution and equality of variance were determined for all datasets. The results for CLD-1 and CLD-4 protein expression required a square root transformation to achieve normal distribution and equality of variance. A two-way analysis of variance (ANOVA) was employed to evaluate the impact of two experimental factors, namely the early stage of CRC and the type of dietary intervention, as well as their interaction. Additionally, Tukey’s post hoc test was utilized to determine the significance of differences among the groups. Statistical significance was established at a p-value of less than 0.05. All graphs were created using GraphPad Prism, version 9.3.1, from GraphPad Software (GraphPad Software Inc., San Diego, CA, USA).

## 5. Conclusions

In this study, we investigated the effects of OBG dietary consumption on gut microbiota, intestinal barrier integrity, and immune profiles in a rat model of AOM-induced early-stage CRC. Our results showed that early-stage CRC altered gut microbiota composition, impaired intestinal barrier integrity, and changed the colon lamina propria lymphocyte profile. The supplementation with 1% OBG showed promising results, increasing the expression of CLD-3 and CLD-4, strengthening the epithelial barrier, and promoting the increase in beneficial butyrate-producing bacteria like *Lachnospiraceae* and *Ruminococcaceae*. However, 3% OBG supplementation had a more complex effect, reducing these beneficial bacteria while increasing *Akkermansia muciniphila* and *Enterococcus faecalis*. It also restores the percentage of Tc and Th lymphocytes in the colon lamina propria and the *Cld1* gene expression, which are altered by the development of CRC. These indicate that the impact of OBG is dose-dependent and may have varying effects at different levels of supplementation and may be related to health conditions. Further research is needed to explore the potential therapeutic role of dietary fibres like oat beta-glucans in CRC prevention and better understand the interactions between microbiota and the host, particularly in cancer conditions.

## Figures and Tables

**Table 1:** Number of ASVs classified to each phylum—all assigned to phylum or are significantly abundant (detected in all groups in most of the samples).

	All Assigned	Highly Abundant
*Bacillota (Firmicutes)*	411 (83.21%)	159 (81.12%)
*Bacteroidota*	27 (5.46%)	14 (7.14%)
*Actinobacteriota*	26 (5.26%)	13 (6.63%)
*Proteobacteria*	19 (3.85%)	
*Verrucomicrobiota*	7 (1.42%)	10 in total
*Cyanobacteria*	2 (0.4%)	(5.11%)
*Deferribacterota*	2 (0.4%)	

**Table 2:** Number of ASVs present in samples of each group.

CRC Groups			Control Groups		
A0	A1	A3	C0	C1	C3
218 ± 22	221 ± 13	203 ± 7	213 ± 35	244 ± 9	220 ± 29

## Data Availability

The data that support the findings of this study are available upon request from the corresponding author.

## Supplementary Materials

The following supporting information can be downloaded at: https://www.mdpi.com/article/10.3390/ijms252413586/s1.
