# Supplementary material for: Oat Beta-Glucans Modulate the Gut Microbiome, Barrier Function, and Immune Responses in an In Vivo Model of Early-Stage Colorectal Cancer
Source: Int J Mol Sci. 2024 Dec 19;25(24):13586. doi: 10.3390/ijms252413586 (PMC11677220; doi:10.3390/ijms252413586)
Supplement: Supplementary file 1 [file ijms-25-13586-s001.zip › Supp Figure S9.pdf]

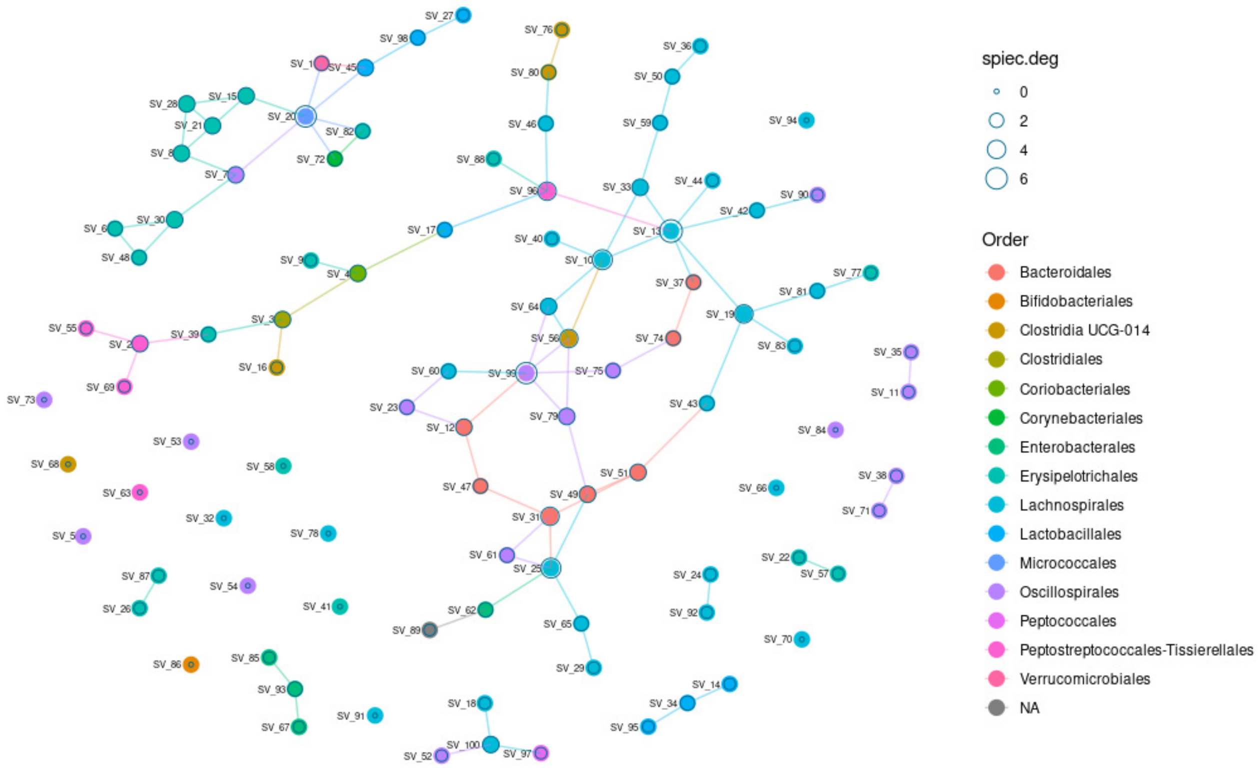


**Figure S9. The network of microbe-to-microbe interactions of 100 most abundant ASVs** with ASV nodes colored by Order lineage. Blue circles indicate the number of interacting species. The top five ASVs with the highest number of interacting species are: **SV_13** ***Blautia pseudococcoides*** (7), **SV_20** *Rothia sp.* (6), **SV_99** *Oscillibacter sp.* (6), **SV_10** ***Muricomes intestini*** (5), and **SV_25** unassigned genus from *Lachnospiraceae* family (5). In brackets the number of directly correlated ASVs is indicated.
