# Supplementary material for: Oat Beta-Glucans Modulate the Gut Microbiome, Barrier Function, and Immune Responses in an In Vivo Model of Early-Stage Colorectal Cancer
Source: Int J Mol Sci. 2024 Dec 19;25(24):13586. doi: 10.3390/ijms252413586 (PMC11677220; doi:10.3390/ijms252413586)
Supplement: Supplementary file 1 [file ijms-25-13586-s001.zip › Supp Figures 1_4.pdf]

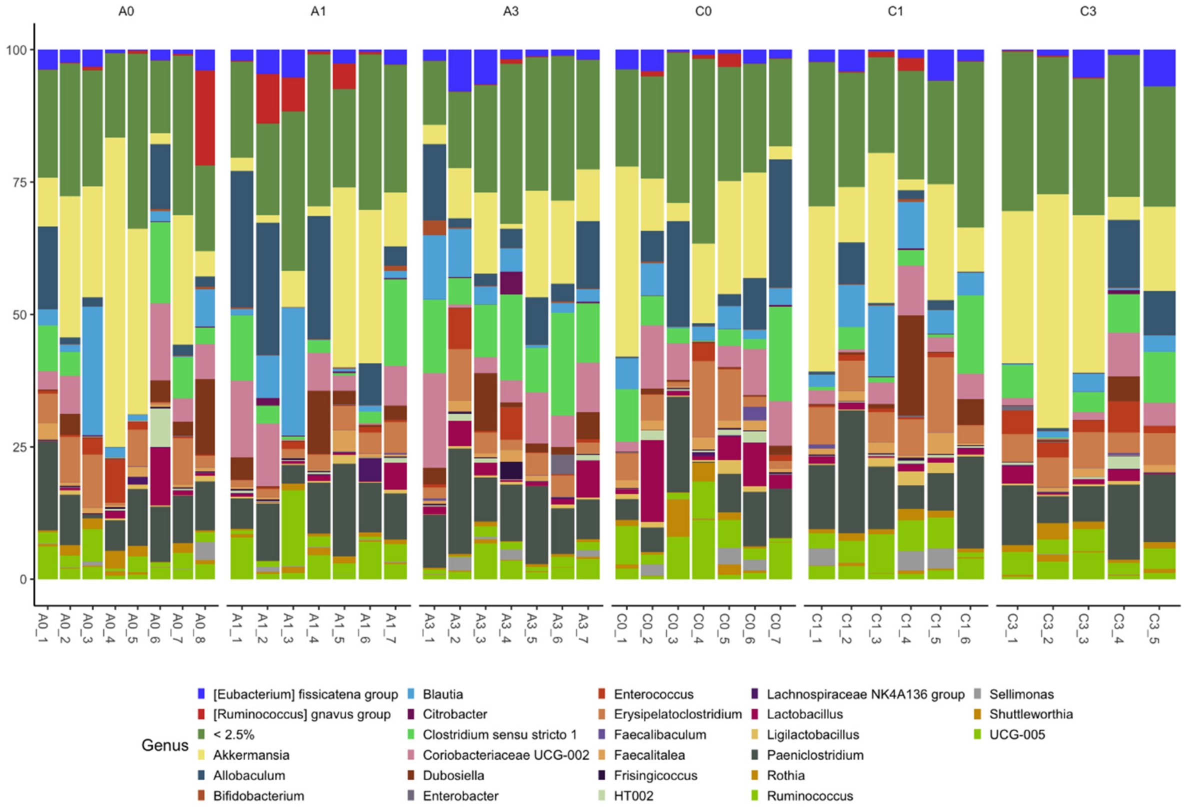

Figure S1. Microbiota composition at taxonomic class-level of genus. Cumulated bar plots represent microbiota composition in each sample; C0 – control rats group fed with diet lacking beta-glucans (n=7); C1 – control rats group fed with diet supplied with 1% of beta-glucans (n=6); C3 - control rats group fed with diet supplied with 3% of beta-glucans (n=5); A0 – CRC model rats group fed with diet lacking beta-glucans (n=8); A1 – CRC model rats group fed with diet supplied with 1% of beta-glucans (n=7); A3 - CRC model rats group fed with diet supplied with 3% of beta-glucans (n=7).

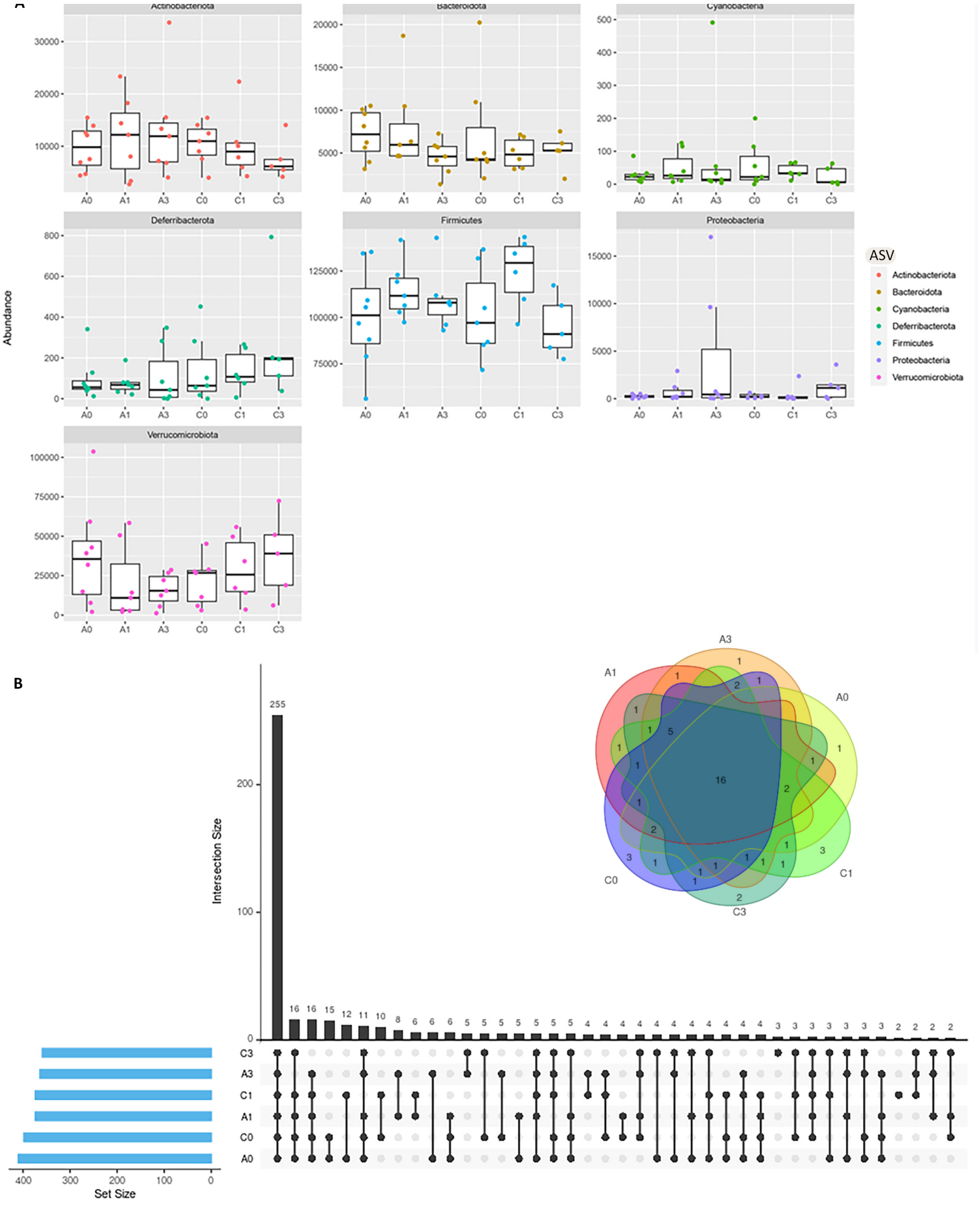

Figure S2. Overview of the gut microbiome composition. A) Bar plots depicting the abundance (counts) of Phyla present in all investigated groups. B) UpSet plot showing the number of detected bacterial species identified in each group and Venn diagram for core species (richness ≥ 0.01, prevalence ≥ 0.95). The number above each bar indicated the amount of the common ASVs (Intersection Size) for the groups marked by black dots on the diagram below the graph. 255 ASVs can be found in all the analysed groups. The Set Size on the left shows the total number of ASVs in each group. The Venn diagram depicts how the core species are distributed in investigated groups – the detailed information about the core

species and their distribution are gathered in the Supp. Table 3.; Group description as in Fig. S1

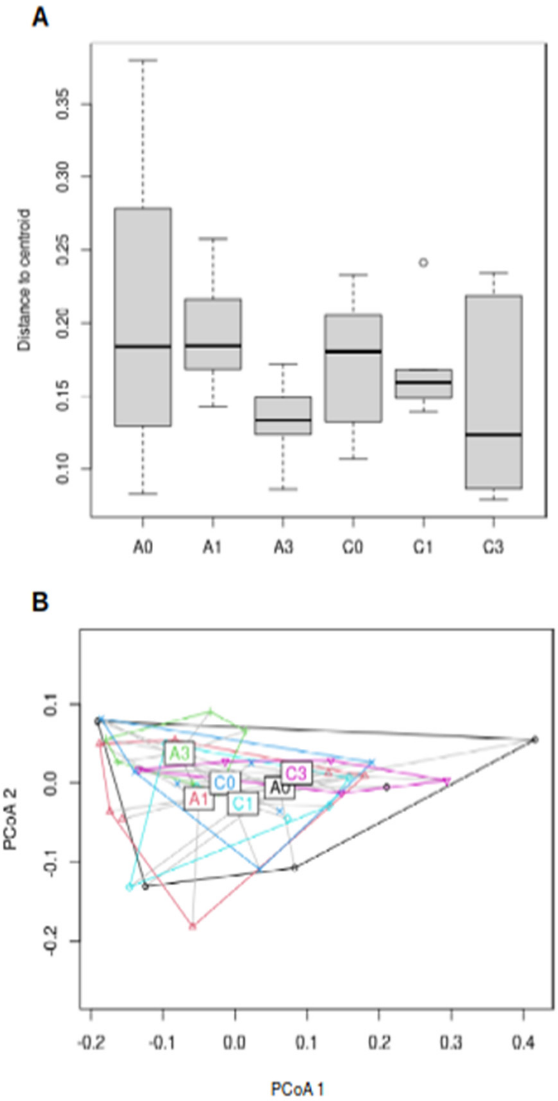

Figure S3. Dissimilarity in community composition between samples quantified by distance or divergence (PERMANOVA analysis results). A) Boxplot of distances from centroid for each condition. B) Graphical representation of data (PCoA plot). Group description as in Fig. S1

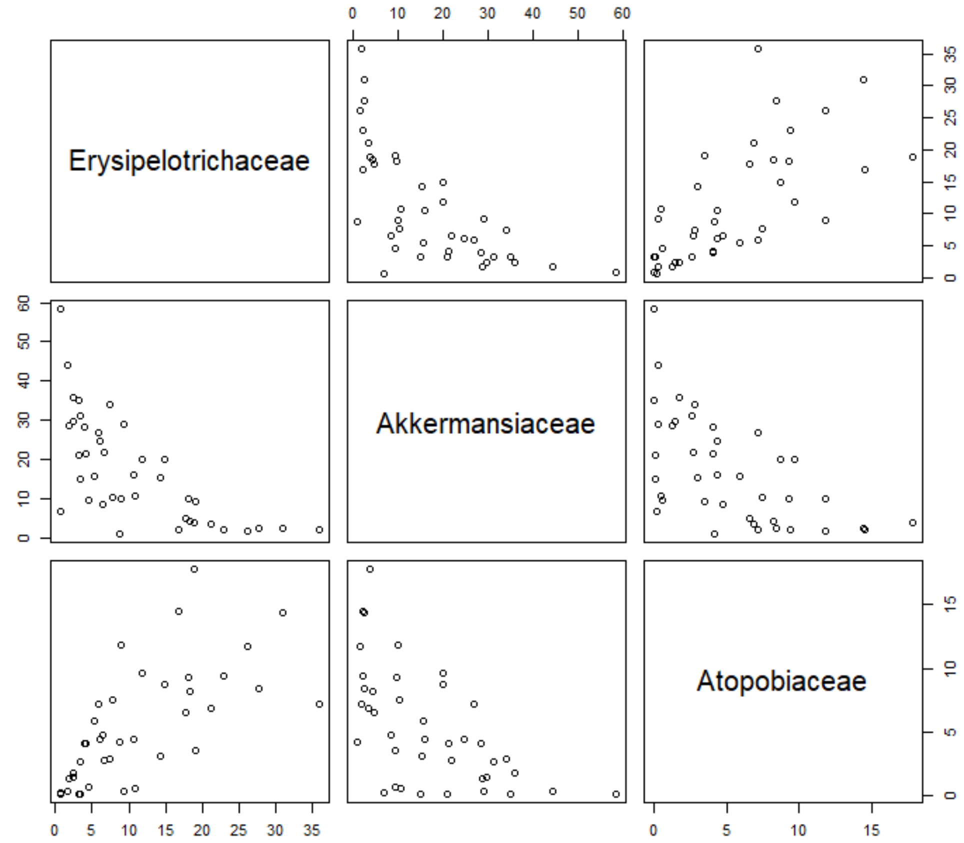

Figure S4. Correlation plot of abundance of *Erysipelotrichaceae*, *Akkermansiaceae* and *Atopobiaceae* families' bacteria in investigated samples. Calculated correlation between *Akkermansiaceae–Atopobiaceae* equals -0.593, between *Erysipelotrichaceae–Akkermansiaceae* equals -0.686, and between *Erysipelotrichaceae–Atopobiaceae* equals 0.681. For all comparisons adj. p-value < 0.001
