# Supplementary material for: Oat Beta-Glucans Modulate the Gut Microbiome, Barrier Function, and Immune Responses in an In Vivo Model of Early-Stage Colorectal Cancer
Source: Int J Mol Sci. 2024 Dec 19;25(24):13586. doi: 10.3390/ijms252413586 (PMC11677220; doi:10.3390/ijms252413586)
Supplement: Supplementary file 1 [file ijms-25-13586-s001.zip › Supp Figures 5_8.pdf]

Figure S5. Abundance of dominating bacteria families in investigated samples. The abundance is expressed as a percentage of the total bacteria abundance in each sample.

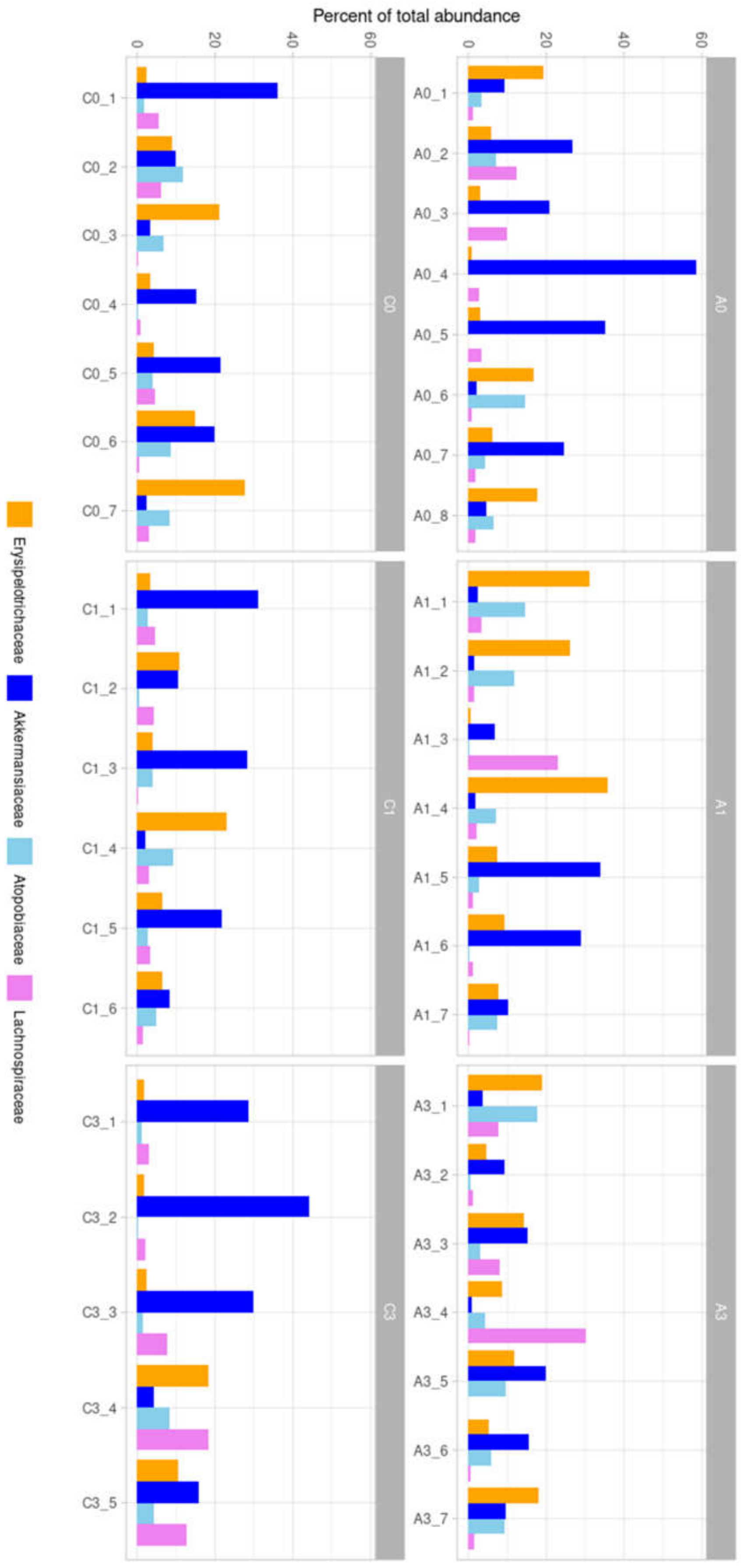

Figure S6. Abundance of bacteria families with known mucolytic bacterias in investigated samples. The abundance is expressed as a percentage of the total bacteria abundance in each sample

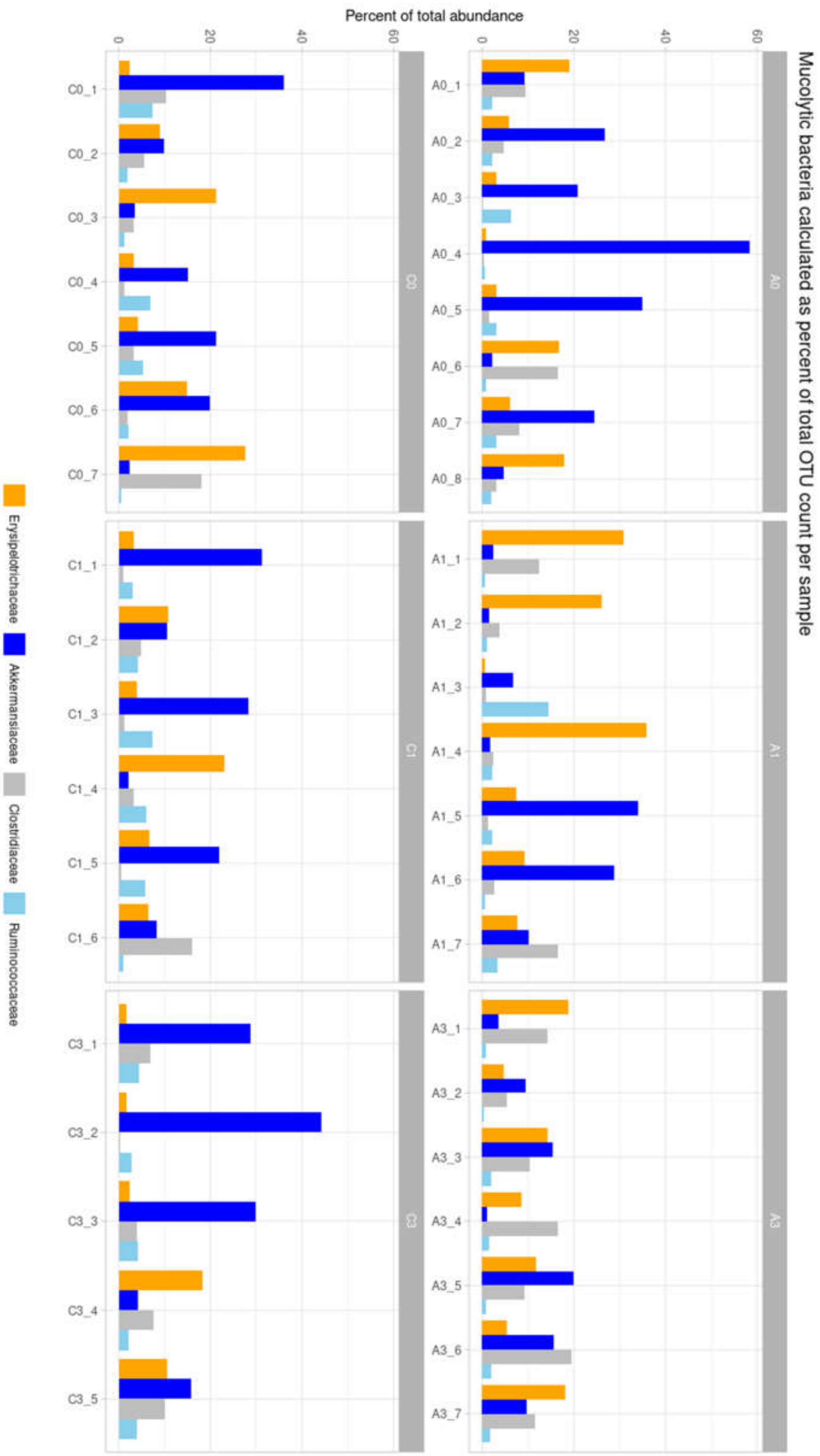

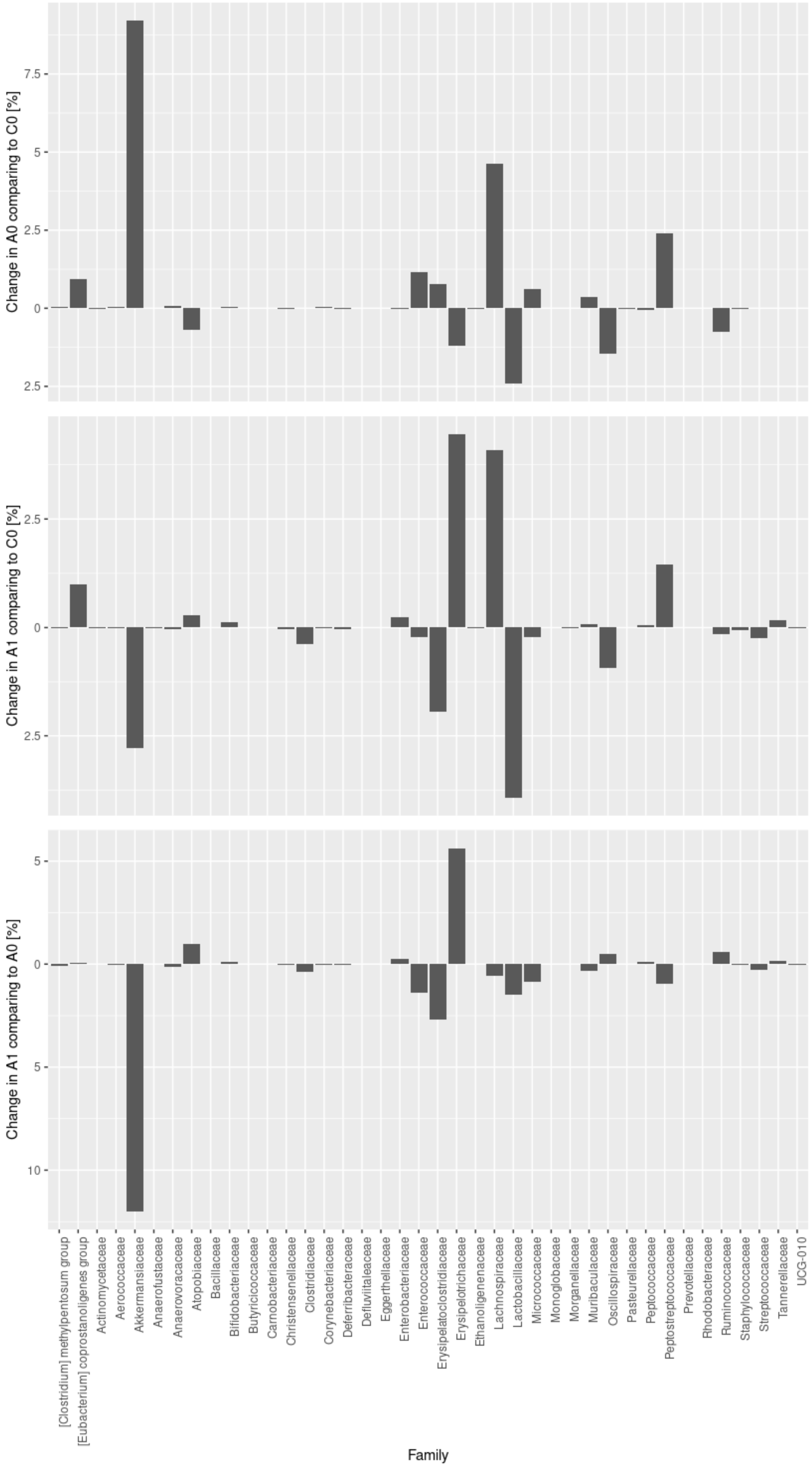

Figure S7. Comparison of change in bacteria families' abundance (in percent) in pairs: A0-C0, A1-C0, and A1-A0.

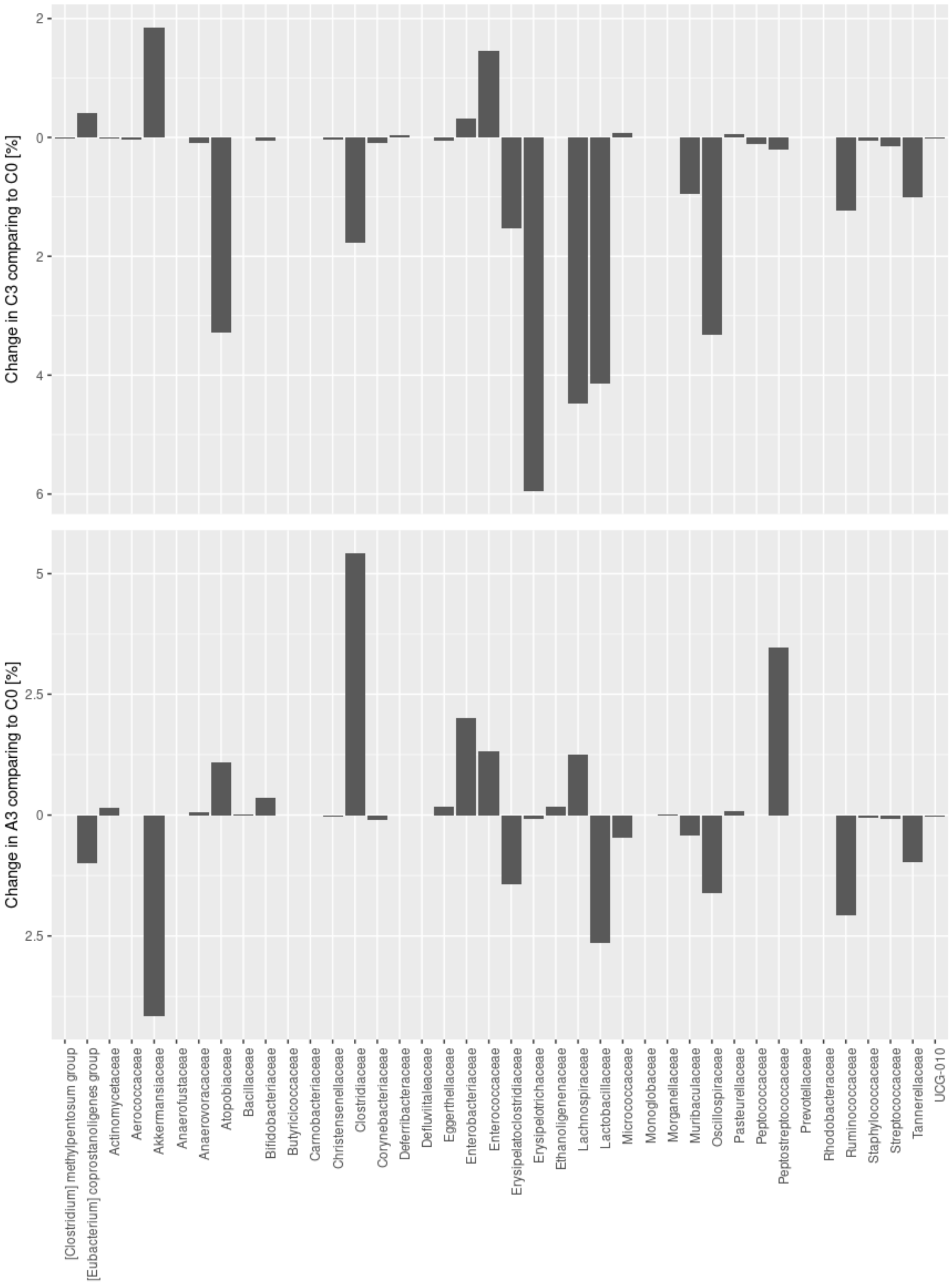

Figure S8. Comparison of change in bacteria families' abundance (in percent) in pairs: C3-C0, and A3-C0.
