# Supplementary material for: Oat Beta-Glucans Modulate the Gut Microbiome, Barrier Function, and Immune Responses in an In Vivo Model of Early-Stage Colorectal Cancer
Source: Int J Mol Sci. 2024 Dec 19;25(24):13586. doi: 10.3390/ijms252413586 (PMC11677220; doi:10.3390/ijms252413586)
Supplement: Supplementary file 1 [file ijms-25-13586-s001.zip › Supp.Table S3.pdf]

Supp. Table S3. Core species and their distribution in investigated groups

Species	ASV	C0	C1	C3	A0	A1	A3
Akkermansia muciniphila	SV_1	+	+	+	+	+	+
Paraclostridium bifermentans	SV_2	+	+	+	+	+	+
Clostridium disporicum	SV_3	+	+	+	**N**	+	+
Muricaecibacterium torontonense	SV_4	+	+	+	**N**	+	+
[Eubacterium] coprostanoligenes group	SV_5	+	+	+	+	+	+
Erysipelatoclostridium ramosum	SV_6	+	+	+	+	+	+
Ruminococcus bromii	SV_7	+	+	+	+	+	+
Allobaculum sp.	SV_8	+	+	**N**	**N**	**N**	+
Dubosiella newyorkensis	SV_9	+	+	+	+	+	+
Muricomes intestini	SV_10	+	+	+	+	+	+
Oscillospiraceae UCG-005	SV_11	+	+	+	+	+	+
Parabacteroides goldsteinii	SV_12	+	+	+	+	+	+
Blautia pseudococcoides	SV_13	+	+	+	**N**	+	+
Lactobacillus johnsonii	SV_14	+	+	+	**N**	+	+
Allobaculum fili	SV_15	+	+	**N**	**N**	**N**	+
Acetivibrio sp.	SV_16	+	+	+	**N**	**N**	+
Enterococcus faecalis	SV_17	**N**	+	+	+	+	+
[Ruminococcus] gnavus group	SV_18	**N**	+	**N**	**N**	+	**N**
Blautia sp.	SV_19	+	+	+	**N**	+	+
Rothia sp.	SV_20	+	+	+	+	+	+
Allobaculum sp.	SV_21	+	**N**	**N**	**N**	**N**	+
Faecalibacterium sp.	SV_22	+	+	**N**	+	+	**N**
[Eubacterium] coprostanoligenes group	SV_23	+	+	+	+	+	+
Lachnospiraceae [unclassified]	SV_25	+	+	+	+	+	+
Ligilactobacillus murinus	SV_27	+	+	+	**N**	+	**N**
Allobaculum sp.	SV_28	**N**	**N**	**N**	**N**	**N**	+
Erysipelatoclostridium sp.	SV_30	**N**	**N**	+	**N**	+	**N**
Muribaculaceae [unclassified]	SV_31	+	+	+	+	+	+
Lachnospiraceae [unclassified]	SV_32	**N**	+	+	+	+	+
Lactobacillus reuteri	SV_34	+	+	**N**	**N**	**N**	**N**
Oscillospiraceae UCG-005	SV_35	**N**	+	+	**N**	+	+
Ruminococcus lactaris	SV_36	+	+	+	+	+	+
Paramuribaculum intestinale	SV_37	+	**N**	+	+	+	**N**
Oscillospiraceae [unclassified]	SV_38	+	+	+	+	+	+
Turicibacter sp.	SV_39	**N**	+	+	**N**	**N**	+
Eisenbergiella massiliensis	SV_42	+	+	+	+	+	+
Lachnospiraceae UCG-001	SV_44	**N**	+	+	+	**N**	**N**
Streptococcus sp.	SV_45	**N**	+	+	**N**	**N**	**N**
Frisingicoccus caecimuris	SV_46	+	+	**N**	+	+	**N**
Muribaculaceae [unclassified]	SV_47	**N**	**N**	+	+	+	**N**
Erysipelatoclostridium sp.	SV_48	**N**	**N**	+	**N**	**N**	**N**
Muribaculaceae [unclassified]	SV_49	+	**N**	+	+	**N**	**N**
Muribaculaceae [unclassified]	SV_51	+	**N**	+	+	**N**	+
Oscillospiraceae UCG-005	SV_52	+	**N**	**N**	**N**	**N**	**N**
Oscillospiraceae NK4A214 group	SV_54	**N**	+	**N**	**N**	**N**	**N**
Romboutsia sp.	SV_55	**N**	**N**	**N**	**N**	+	+
Faecalibacterium sp.	SV_57	+	**N**	**N**	**N**	**N**	**N**
Longibaculum muris	SV_58	**N**	+	**N**	**N**	**N**	**N**
Clostridia UCG-014	SV_68	+	**N**	+	**N**	**N**	**N**
Paeniclostridium sp.	SV_69	**N**	**N**	+	**N**	**N**	**N**
Oscillospiraceae [unclassified]	SV_71	**N**	**N**	**N**	+	**N**	**N**
Ruminococcaceae [Incertae Sedis]	SV_73	**N**	+	**N**	**N**	**N**	**N**
Clostridia [unclassified]	SV_89	+	**N**	**N**	**N**	**N**	**N**

"+" - core species or "N" – not a core species, in selected group
